# pVHL-mediated regulation of the anti-angiogenic protein thrombospondin-1 decreases migration of Clear Cell Renal Carcinoma Cell Lines

**DOI:** 10.1038/s41598-020-58137-w

**Published:** 2020-01-24

**Authors:** Javier Sevilla-Montero, Raquel Bienes-Martínez, David Labrousse-Arias, Esther Fuertes-Yebra, Ángel Ordóñez, María J. Calzada

**Affiliations:** 1 Biomedical Research Institute La Princesa Hospital (IIS-IP), Department of Medicine, School of Medicine, Autónoma University of Madrid, Madrid, Spain; 2 Research Unit, Hospital Santa Cristina, Biomedical Research Institute Princesa (IIS-IP), Madrid, Spain

**Keywords:** Extracellular matrix, Cancer

## Abstract

Thrombospondin-1 (TSP-1) is a multifunctional matrix protein with antitumor activities due in part to its ability to inhibit angiogenesis, which in turn contributes to determine the fate of many tumours. Previous studies have shown that TSP-1 expression supports normal kidney angiostasis, and decreased TSP-1 levels contribute to the angiogenic phenotype of renal cell carcinomas (RCC). The loss of the von Hippel-Lindau tumour suppressor gene (*VHL*) in these tumours favours stabilization of the Hypoxia Inducible Factors (HIF), which in turn contribute to adapt tumour cells to hostile environments promoting tumour progression. However, HIF-independent regulation of certain genes might also be involved. We have previously shown that TSP-1 is regulated in hypoxia in clear cell RCC (ccRCC) in a HIF-independent manner; however, the effect of VHL protein (pVHL) on TSP-1 expression has not been evaluated. Our results proved that pVHL loss or mutation in its alpha or beta domain significantly decreased TSP-1 levels in ccRCC in a HIF-independent manner. Furthermore, this regulation proved to be important for ccRCC cells behaviour showing that decreased TSP-1 levels rendered ccRCC cells more migratory. This data substantiates a unique regulation pattern for TSP-1 in a pVHL-dependent manner, which may be relevant in the aggressiveness of ccRCC.

## Introduction

Dysregulation of the von Hippel-Lindau (*VHL*) gene is closely associated with clear cell renal cell carcinoma (ccRCC), being this one of the most common features in patients with the Von Hippel-Lindau disease^1^. This disease is an autosomal dominant hereditary cancer syndrome caused by germ line mutations or deletions in the *VHL* tumour suppressor gene^2^. Several pieces of evidence have implicated this gene as a gatekeeper gene in the pathogenesis of RCC, these including *VHL* gene mutations in most primary sporadic renal cell carcinomas (RCCs) and the development of renal cysts in *Vhl* conditional knockout mice^3–7^. VHL protein (pVHL) is the substrate recognition component of a multi-subunit complex with E3 ubiquitin ligase activity that also comprise elongin B, elongin C, Cul2 and RBx1^8,9^. This protein complex is also a central component in the oxygen-sensing machinery involved in the proteasome-mediated degradation of the hypoxia-inducible factor (HIF)-α^10,11^. Therefore, *VHL* loss or certain mutations in this tumour suppressor gene lead to the stabilization of HIF in normoxia^12^ which in turn promotes the transcription of HIF target genes, these including angiogenic factors like vascular endothelial growth factor A (VEGF A)^13^. Although the high expression of VEGF may provide an explanation for the high vascularization of these pVHL-negative tumours, this alone is not sufficient to promote the growth of RCC xenografts and additional events may be involved. In this respect, other pVHL HIF-independent functions have been shown to be required and help to explain why loss of pVHL leads to renal cancer^14^. In particular, it is interesting to highlight the pVHL-mediated regulation of intercellular junctions and extracellular matrix homeostasis and its contribution to the growth and progression of RCC^15–17^. In addition, negative regulation of anti-angiogenic factors may also contribute to promote growth of RCC xenografts; however, their role in the progression of these carcinomas has been largely ignored. One of these factors is the multimeric and multifunctional matricellular protein thrombospondin-1 (TSP-1). This protein belongs to a family of five extracellular matrix proteins with similar structures^18,19^ and is the first identified angiogenesis inhibitor^20,21^. Furthermore, its expression is critical for the maintenance of the anti-angiogenic microenvironment in a variety of experimental tumours and metastases, such as breast, brain, colon, bladder and skin^22–27^.

TSP-1 modulates cell behaviour by altering cell adhesion, motility, proliferation, survival and growth of many cell types *in vitro*, gene expression, and differentiation^28,29^. Additionally, TSP-1 expression is frequently lost during malignant transformation due to regulation of its expression by oncogenes, tumour suppressor genes and hypermethylation^27,30,31^. These properties have attracted interest in this matricellular protein as a potential regulator of tumour growth and metastasis.

Our results demonstrate that pVHL expression positively regulates TSP-1 protein levels, and this regulation is not at the transcriptional level since we did not observe regulation of TSP-1 mRNA levels. In addition, we also demonstrate that TSP-1 regulation by pVHL is a HIFα−independent event. Finally, we analysed the relevance of TSP-1 regulation in the aggressiveness of these carcinoma cells and found that decreased TSP-1 expression rendered ccRCC cells more migratory.

## Results

### TSP-1 levels are diminished in ccRCC cells lacking pVHL

**Figure 1:**
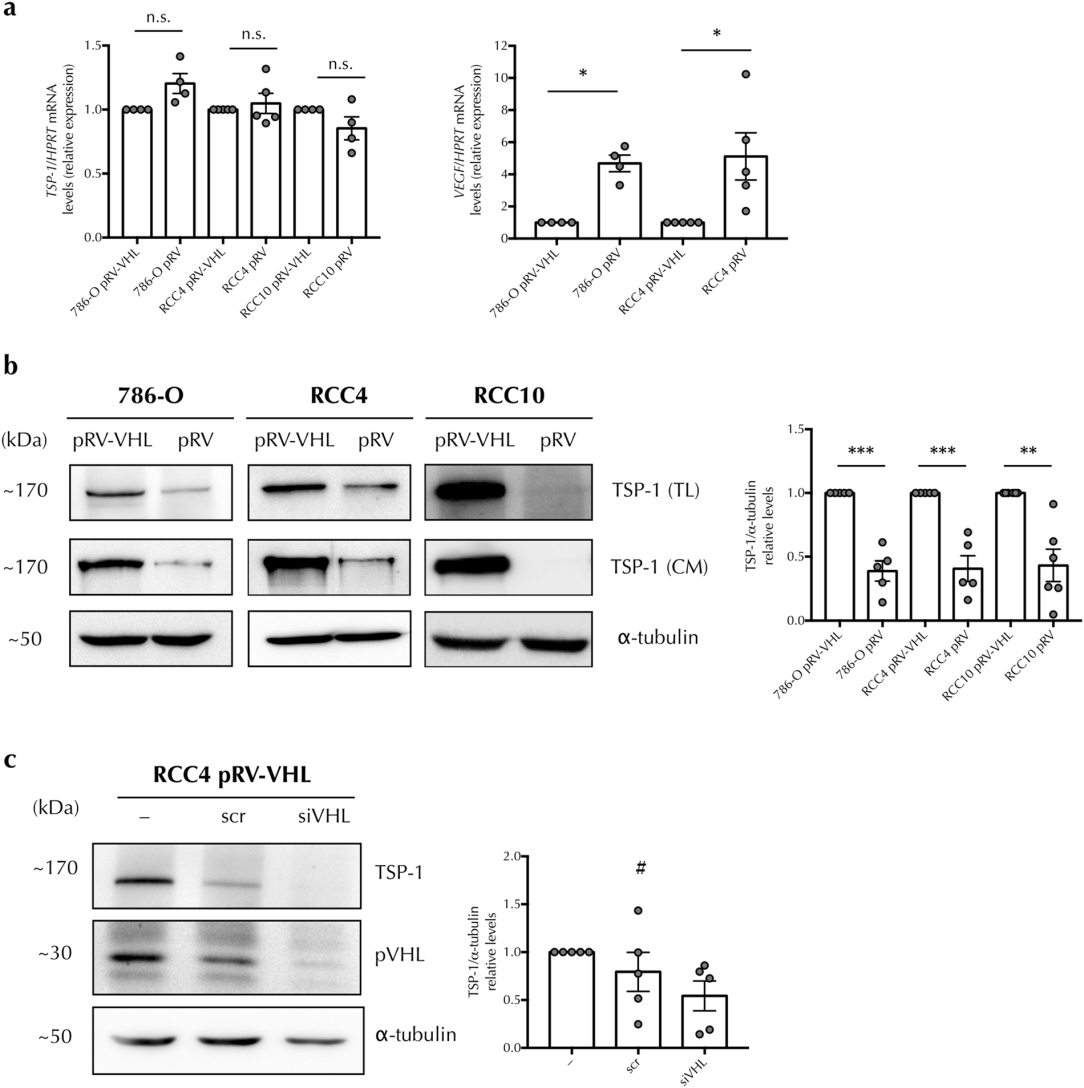
TSP-1 levels are diminished in ccRCC cells lacking pVHL. (**a**) Quantitative RT–PCR analysis was performed to determine *TSP-1* and *VEGF* mRNA expression levels from pVHL-positive (pRV-VHL) and negative (pRV) 786-O, RCC4 and RCC10 cell lines. mRNA levels are expressed as fold change over pVHL-positive cells, normalized with *HPRT* as housekeeping gene and presented as mean ± SEM, n = 4–5. Statistical comparisons between different pairs were made using one-sample t-test (n.s. = non-significant, *P < 0.05). (**b**) Protein levels from total cell lysates (TL) and conditioned media (CM) from pVHL-positive and negative cells were determined by western blot probed against TSP-1 and α-tubulin as loading control. Representative images and band quantifications by densitometry are shown and presented as mean ± SEM, n = 5–6. Statistical comparisons between different pairs were made using one-sample t-test (**P < 0.01, ***P < 0.005). (**c**) pVHL-positive RCC4 cells were transiently transfected with control (scr) or pVHL-specific siRNA (siVHL). Protein levels from total cell lysates from non-transfected (–), scr or pVHL-specific (siVHL) siRNA-transfected pVHL-positive RCC4 cells were determined by western blot probed against TSP-1, pVHL and α-tubulin as loading control. Representative images and band quantifications by densitometry are shown and presented as mean ± SEM, n = 5. Statistical comparisons between different conditions were made using one-way ANOVA test followed by linear trend *post-hoc* test (^#^*P-*trend *<* 0.05). Full-length blots are presented in Supplementary Fig. S2.

As a tumour suppressor protein, pVHL plays an important role in the regulation of extracellular matrix homeostasis, an important event in the growth and progression of renal cell carcinomas^17,32–36^. Considering this, we asked whether pVHL could also regulate TSP-1. We analysed TSP-1 mRNA and protein expression levels in several ccRCC cell lines (786-O, RCC4 and RCC10). TSP-1 mRNA levels did not differ significantly between pVHL positive and negative clones, although VEGF mRNA showed the expected up-regulation of gene expression in the absence of pVHL (Fig. 1a). In contrast, TSP-1 protein levels were significantly decreased in total cell lysates from the different ccRCC parental cell lines (786-O, RCC4 and RCC10) compared to the clones stably expressing pVHL (Fig. 1b). Given that TSP-1 is a glycoprotein that is actively secreted into the medium, we also analysed whether decreased protein levels found in our total cell lysates were due to an increased rate of TSP-1 secretion. TSP-1 levels in conditioned media were correspondingly decreased in cells lacking pVHL compared to the respective pVHL positive cells (Fig. 1b). Thus, total TSP-1 protein levels are decreased in cell lines lacking pVHL. In order to confirm that the decrease of TSP-1 levels was due to the lack of pVHL and not to secondary alterations occurring during passage, we knocked down *VHL* by siRNA, and the degree of silencing was quantified by western blot. *VHL* interference led to a marked decrease of TSP-1 protein levels, similar to those in pVHL negative cells (Fig. 1c).

### pVHL mutations in its alpha or beta domain decrease TSP-1 levels

**Figure 2:**
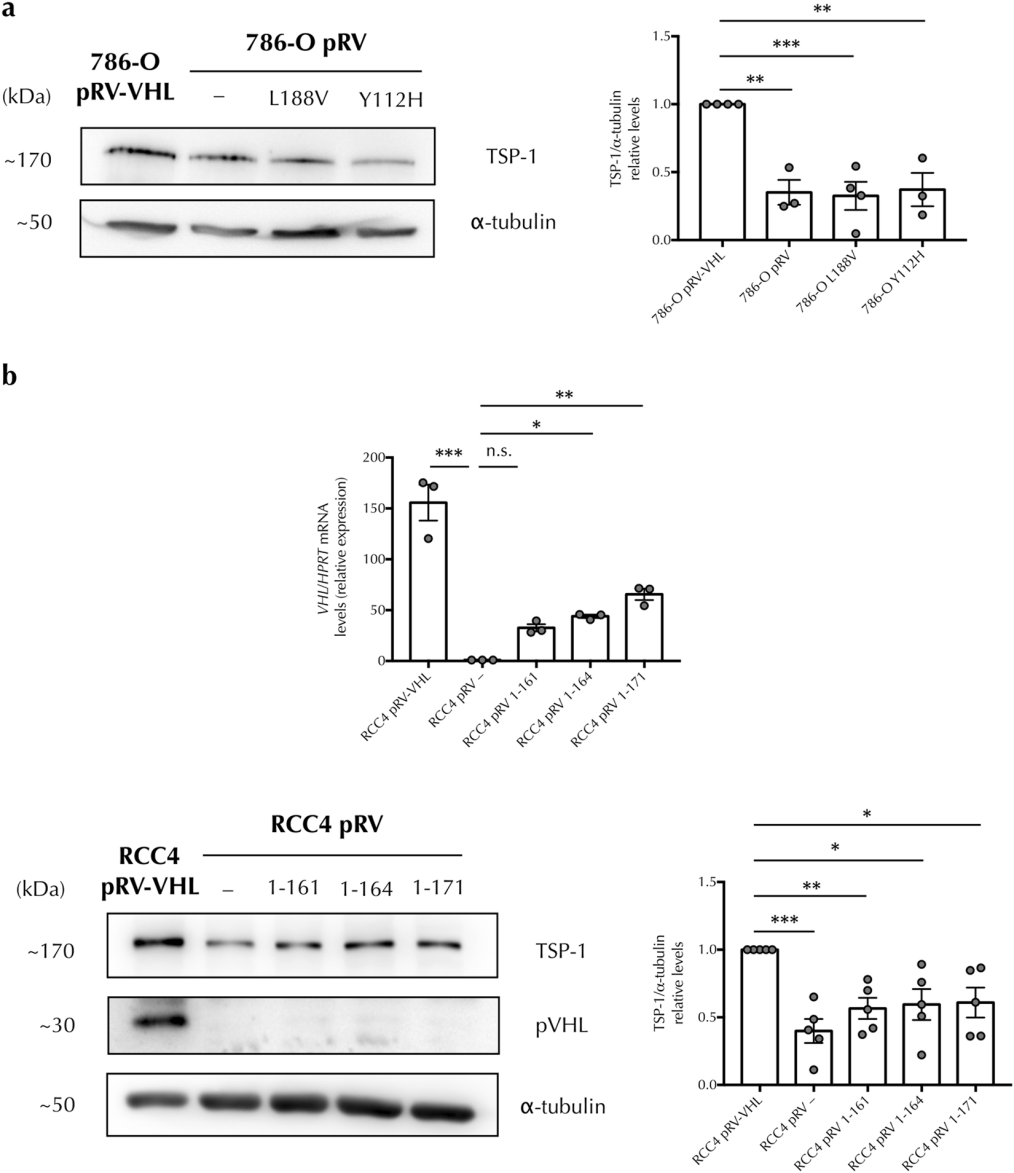
pVHL mutations in its alpha or beta domain decrease TSP-1 levels. (**a**) Protein levels from pVHL-positive, pVHL-negative, and pVHL-mutant-expressing (L188V or Y112H) 786-O cells were determined by western blot probed against TSP-1 and α-tubulin as loading control. Representative images and band quantifications by densitometry are shown and presented as mean ± SEM, n = 3. Statistical comparisons between different conditions were made using one-way ANOVA test followed by Bonferroni’s *post-hoc* test (**P < 0.01, ***P < 0.005). (**b**) Quantitative RT–PCR analysis was performed to determine *VHL* mRNA expression from pVHL-positive, pVHL-negative and truncated pVHL-mutant-expressing (1–161, 1–164 or 1–171) RCC4 cells. mRNA levels are expressed as fold change over pVHL-negative cells, normalized with *HPRT* as housekeeping gene and presented as mean ± SEM, n = 3 (upper panel). Protein levels from pVHL-positive, pVHL-negative and truncated pVHL-mutant-expressing (1–161, 1–164 or 1–171) RCC4 cells were determined by western blot probed against TSP-1, pVHL and α-tubulin as loading control. Representative images and band quantifications by densitometry are shown and presented as mean ± SEM, n = 5 (lower panels). Statistical comparisons between different conditions were made using one-way ANOVA test followed by Bonferroni’s *post-hoc* test (n.s. = non-significant, *P < 0.05, **P < 0.01, ***P < 0.005). Full-length blots are presented in Supplementary Fig. S2.

In order to gain further proof of the role of pVHL in TSP-1 regulation we also used 786-O sublines stably transfected with well-characterized naturally occurring mutant forms of pVHL in its alpha (L188V) or its beta domain (Y112H). Our results indicated that TSP-1 protein levels were decreased in both mutants to levels resembling those in pVHL negative cells (Fig. 2a). Additionally, we generated different pVHL mutations that produced other truncated forms in its alpha domain (1–161, 1–164 and 1–171) and tested for *VHL* transcripts expression (Fig. 2b, upper panel). TSP-1 levels were similarly decrease with all these mutants compared with the wild type pVHL (Fig. 2b, lower panels). Taken together these data demonstrate that in ccRCC cell lines TSP-1 protein levels are regulated in a pVHL-dependent manner and, most importantly, both, its alpha and beta domains are critical for the regulation of TSP-1 protein.

### TSP-1 regulation in ccRCC cell lines is not mediated by its interaction with pVHL

**Figure 3:**
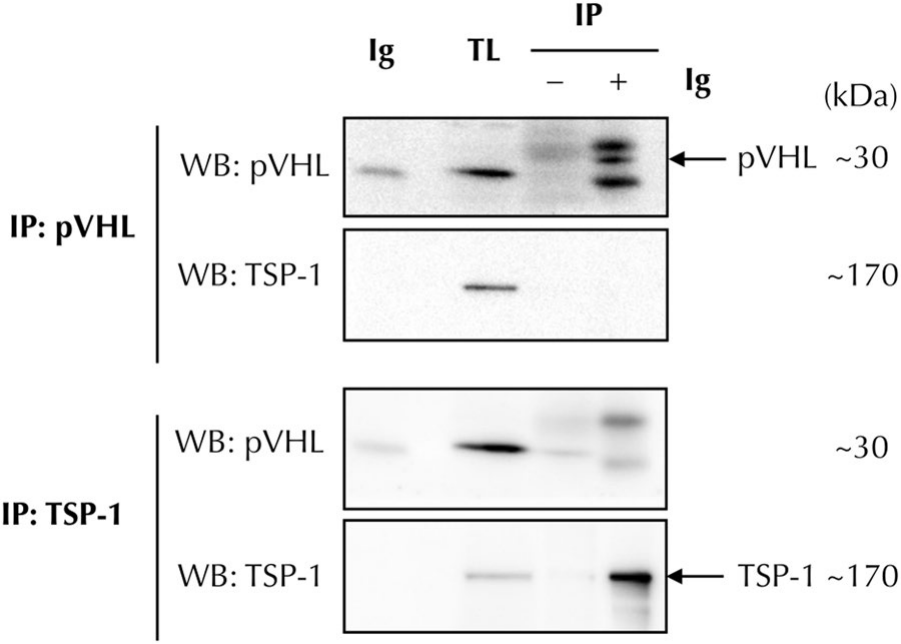
TSP-1 regulation in ccRCC cell lines is not mediated by its interaction with pVHL. Total cell lysates from pVHL-positive cells were immunoprecipitated (IP) to study the association of pVHL and TSP-1 proteins. Immunoprecipitation was carried out with antibodies against pVHL (upper panels) or TSP-1 (lower panels), and protein levels were determined afterwards by western blot probed against pVHL and TSP-1. Additional lanes showing the signal from the antibodies used in the immunoprecipitation (Ig) and from total lysates (TL), are also included in the western blot images. Full-length blots are presented in Supplementary Fig. S2.

It has been previously shown that pVHL binds to proteins increasing their stability, without causing variations in their mRNA^37,38^. Taking this into account and the results with TSP-1 shown above, we considered analysing whether the decrease in protein in ccRCC lines could be related to the lack of interaction between pVHL and TSP-1. To this aim, we carried out immunoprecipitation experiments. After analysing the results by western blotting, no bands corresponding to TSP-1 were observed in the pVHL immunoprecipitates. Likewise, we did not observe bands corresponding to pVHL when precipitating the lysates with anti-TSP-1 antibodies (Fig. 3). Therefore, these results indicate that the regulation of TSP-1 in ccRCC does not depend on its direct interaction with pVHL.

### TSP-1 decrease in pVHL negative cells is HIF independent

**Figure 4:**
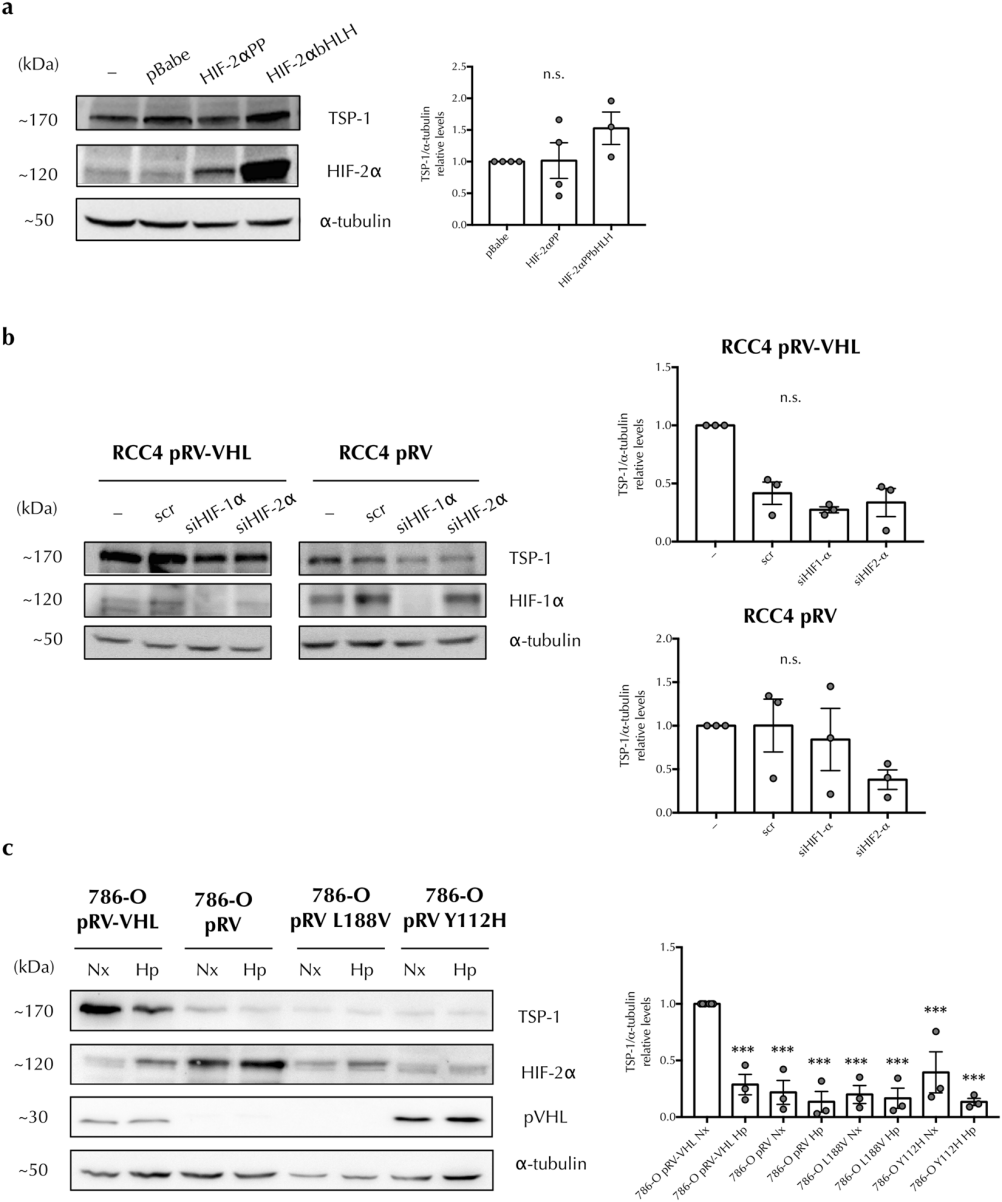
TSP-1 decrease in pVHL-negative cells is HIF-independent. (**a**) Protein levels from control or HIF-2α-mutant-expressing pVHL-positive 786-O cells (empty vector pBabe, HIF-2αPP or HIF-2αbHLH mutants) were determined by western blot probed against TSP-1, HIF-2α and α-tubulin as loading control. Representative images and band quantifications by densitometry are shown and presented as mean ± SEM, n = 3–4. Statistical comparisons between different conditions were made using one-way ANOVA test followed by Bonferroni’s *post-hoc* test (n.s. = non-significant). (**b**) Protein levels from non-transfected (–), scr, HIF-1α-specific (siHIF-1α) or HIF-2α-specific (siHIF-2α) siRNA-transfected pVHL-positive and pVHL-negative RCC4 cells were determined by western blot probed against TSP-1, HIF-1α and α-tubulin as loading control. Representative images and band quantifications by densitometry are shown and presented as mean ± SEM, n = 3. Statistical comparisons between different conditions were made using one-way ANOVA test followed by Bonferroni’s *post-hoc* test (n.s. = non-significant). (**c**) Protein levels from control pVHL-positive, pVHL-negative, L188V and Y112H pVHL-mutant-expressing 786-O cells cultured under normoxia (Nx, 21% O_2_) or hypoxia (Hp, 1% O_2_) for 24 hours were determined by western blot probed against TSP-1, HIF-2α, pVHL and α-tubulin as loading control. Representative images and band quantifications by densitometry are shown and presented as mean ± SEM, n = 3. Statistical comparisons between different conditions were made using one-way ANOVA test followed by Bonferroni’s *post-hoc* test (***P < 0.005 with respect to the levels of 786-O cells cultured under normoxia). Full-length blots are presented in Supplementary Fig. S2.

VHL best-known function is the regulation of HIF-α proteolytic degradation^12,39^. Given that in the absence of pVHL these factors are stabilized, we asked whether TSP-1 decreased protein levels in pVHL negative cells were a HIF-mediated effect. To this aim we infected wild-type pVHL-transfected 786-O cells, which only express HIF-2α, with a retrovirus encoding the empty vector (pBabe), a constitutively active form of HIF-2α (HIF-2αpp), or with a mutant lacking transcriptional activity (HIF-2αbHLH)^16^. Western blots quantification from total cell lysates showed no modification on TSP-1 protein levels in cells infected with the constitutively active form of HIF2α (Fig. 4a) indicating that this factor was not involved in TSP-1 protein decrease in the absence of pVHL. To further confirm these results we knocked down the expression of HIF1α or HIF2α in the RCC4 cell line, using specific siRNAs. Interestingly, when we knocked down them in both pVHL positive and negative cells we only observed a slight decrease but never a TSP-1 protein recovery (Fig. 4b). Additionally, HIF-independent regulation of TSP-1 in ccRCC cells was also confirmed in cells expressing the L188V or the Y112H mutant forms of pVHL, the first reported to regulate HIF normally although it is defective in promoting the assembly of fibronectin extracellular matrix^40,41^. Our results demonstrated that despite HIF-2α levels were low in normoxia in this mutant cells, TSP-1 levels were similarly decreased in normoxia or hypoxia in both, the pVHL L188V and Y112H mutants to levels resembling those in pVHL negative cells (Fig. 4c). Taken together these data demonstrate that TSP-1 decrease in ccRCC pVHL negative cell lines is independent of HIF.

### Intercellular junction integrity in pVHL-negative RCC cells is dependent on TSP-1 levels

**Figure 5:**
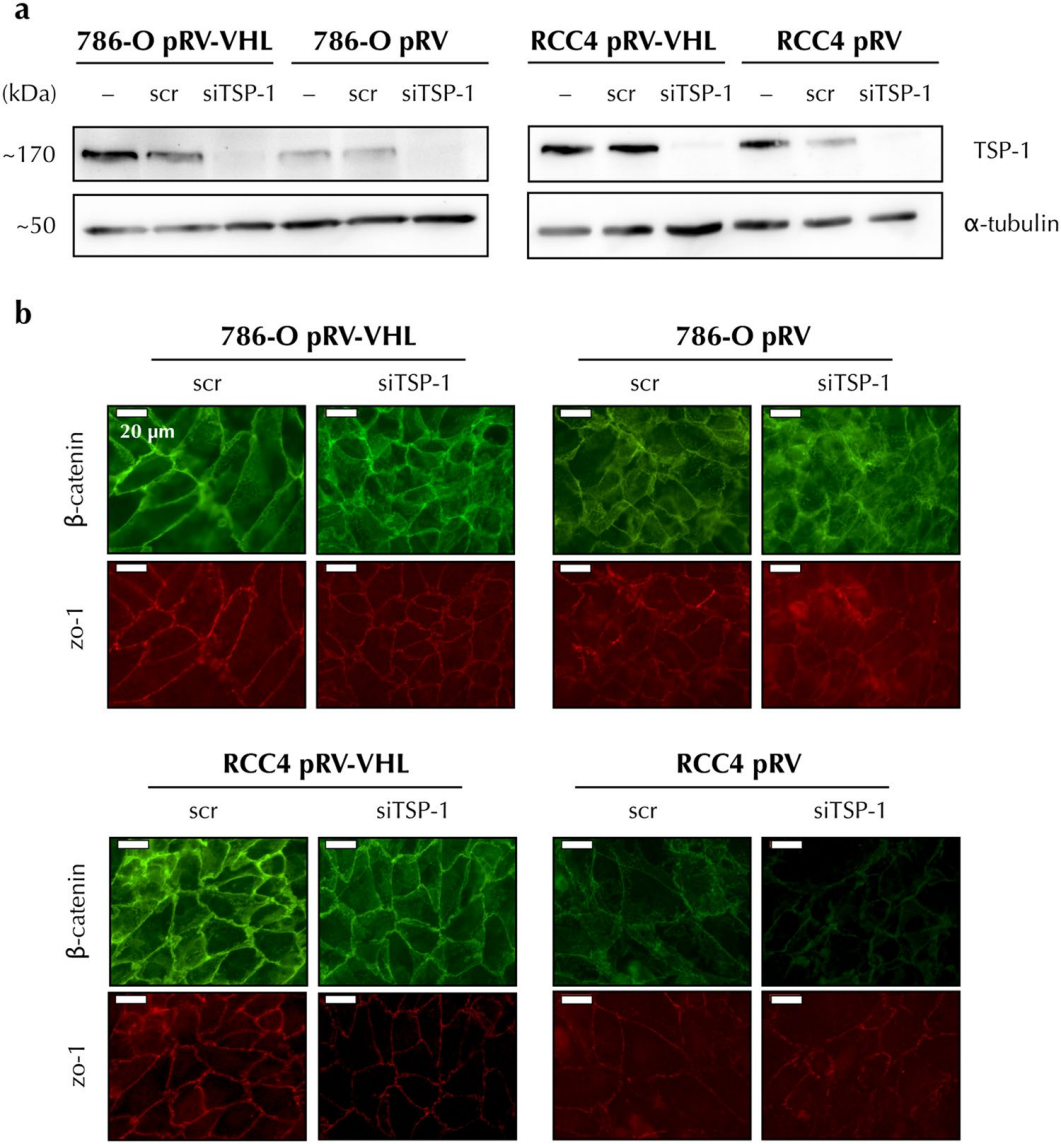
Intercellular junction integrity in pVHL-negative RCC cells is dependent on TSP-1 levels. (**a**) Protein levels from non-transfected (−), scr or TSP-1-specific (siTSP-1) siRNA-transfected pVHL-positive and negative 786-O and RCC4 cells were determined by western blot probed against TSP-1 and α-tubulin as loading control. Full-length blots are presented in Supplementary Fig. S2. (**b**) Immunofluorescence of scr or TSP-1-specific (siTSP-1) siRNA-transfected pVHL-positive and negative 786-O and RCC4 cells was used to visualize β-catenin (green) and ZO-1 (red) in adherens and tight cell junctions. Representative images from three independent experiments are shown.

Our previously published results demonstrate a defective distribution of intercellular junctions in ccRCC, which are restored by the expression of pVHL. Furthermore, these effects are independent on pVHL-mediated regulation of HIF^16^. Hence, we asked whether the decreased levels of TSP-1 in these cells were involved in maintaining intercellular adhesion in ccRCC lines. To analyse this we knocked down this protein in pVHL expressing 786-O and RCC4 cell lines (Fig. 5a) and performed double immunofluorescence staining and microscopy analysis of the junctional proteins β-catenin and zonulin (ZO)-1. Our results showed that pVHL expressing cells with TSP-1 decreased levels displayed an irregular immunofluorescent staining pattern of β-catenin and ZO-1 distribution, thus indicating that pVHL-dependent restoration of normal intercellular junctions was affected by the levels of TSP-1 in these cells (Fig. 5b). These results suggested that both phenomena, pVHL loss and decreased TSP-1 levels in ccRCC, might be linked.

### TSP-1 expression regulates RCC cell migration and invasion

**Figure 6:**
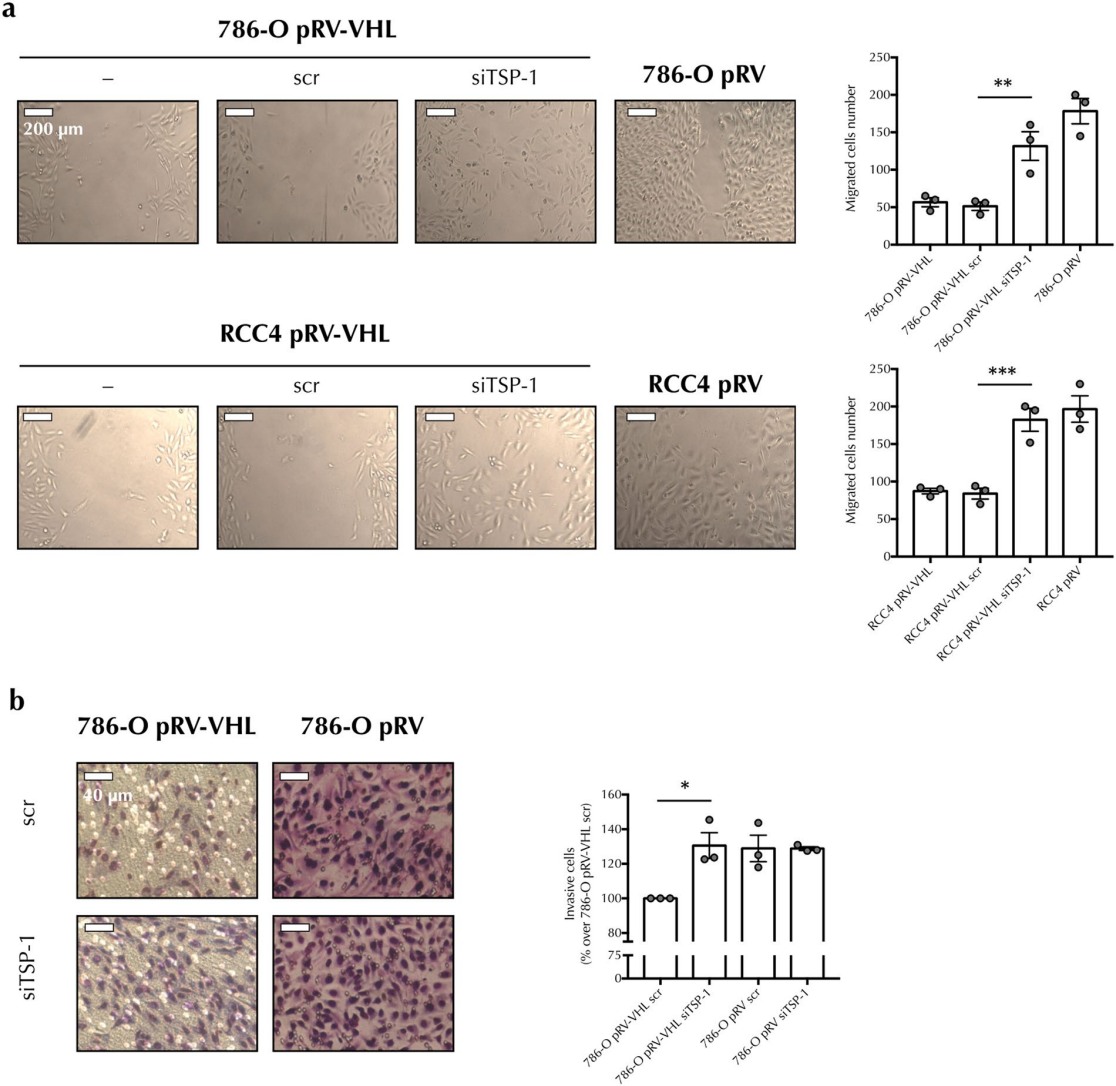
TSP-1 expression regulates RCC cell migration and invasion. (**a**) Non-transfected (–), scr or TSP-1-specific (siTSP-1) siRNA-transfected pVHL-positive and negative 786-O and RCC4 cells were seeded on 24-well plates and experimental wounds were made with a pipette tip across the central line of each well. Cells between the scraped edges were counted 16 hours later. Representative phase contrast images are shown. Data from cell quantification are presented as mean ± SEM, n = 3. Statistical comparisons between different conditions were made using one-way ANOVA test followed by Bonferroni’s *post-hoc* test (**P < 0.01, ***P < 0.005). (**b**) Transwell® assays were used to analyse cell invasion through collagen matrices to FBS as chemoattractant. Scr or TSP-1-specific (siTSP-1) siRNA-transfected pVHL-positive and negative 786-O cells were added on top of type I collagen membranes and migrated cells were stained 16 hours later. Representative images of each condition are shown. Data are presented as mean ± SEM, n = 3. Statistical comparisons between different conditions were made using one-way ANOVA test followed by Bonferroni’s *post-hoc* test (*P < 0.05).

It has been shown that secretion of angiogenic factors stimulates RCC cell migration, although the role of endogenous angiogenic inhibitors such as TSP-1 in autocrine regulation of RCC cells remains unexplored. On the other hand, we have previously described that hypoxia-mediated suppression of TSP-1 promotes migration in ccRCC^42^. Considering this, we asked whether decreased expression of TSP-1 in the absence of pVHL also contributed to RCC cell motility and invasion. To analyse this, we first performed wound healing assays to compare motility between pVHL positive and negative cells. Figure 6a shows a decreased motility of pVHL expressing cells after 16 h compared to pVHL negative cells. To ascertain whether TSP-1 decrease in pVHL negative cells was involved in cell migration we compared migration rates in these cells with pVHL expressing ccRCC cells transfected with scramble or a specific TSP-1 siRNA. As shown in Fig. 6b, pVHL positive cells transfected with TSP-1 siRNA showed increased motility, evidenced by the increased number of cells in the wounded area, compared to cells transfected with a scramble siRNA, whereas the migration was similar to that of pVHL negative cells. In addition, the TSP-1 siRNA-transfected cells were also analysed for their ability to invade through a type I collagen matrix using a Transwell® insert assay system. As a chemoattractant we used medium with 10% FBS or serum-free medium as a negative control. None of the cell lines migrated towards the serum-free media control (data not shown). However, when stimulated with serum, pVHL positive cells transfected with siRNA to TSP-1 showed a significant increase of cell migration/invasion compared to cells transfected with scramble siRNA. Furthermore, this was similar to that of pVHL negative cells (Fig. 6c). Taken together these results indicated that the lack of pVHL in ccRCC promotes an increase of cell migration through decreasing TSP-1 levels.

## Discussion

Renal cell carcinoma is the most common malignant tumour of the adult kidney. Nowadays, advanced renal cell carcinoma is commonly treated with VEGF or mammalian target of rapamycin inhibitors^43^. However, therapies targeting VEGF must be cautiously considered^44^. Therefore, a deeper understanding of the molecular events that occur in these tumours will improve the design of more effective therapies. Our studies in different RCC cell lines indicated that the absence of the tumour suppressor gene pVHL induced a decrease in the expression of the tumour and angiogenesis inhibitor TSP-1. This regulation proved to be important for the RCC cell behaviour and correlated with an increased migratory and invasive potential. Interestingly, our previous studies demonstrate that stabilization of HIFα in ccRCC is not sufficient to restore neither a differentiated epithelial phenotype nor proper intercellular junction assembly^16^. In this paper, we demonstrate that the integrity of intercellular junctions in pVHL-negative RCC cells is dependent on TSP-1 levels. In this respect, previous reports have shown that TSP-1 is important in maintaining normal kidney angiostasis and its expression inversely correlates with microvascular density, proliferation and progression in RCC^42,45–47^; however, none of them have addressed whether the lack of pVHL in these tumours affects TSP-1 expression. In this line, Veliceasa *et al*. found that TSP-1 secretion is impaired in RCC compared with normal renal epithelium due to protein misfolding caused by changes in calcium uptake^46^. However, this cannot explain the decreased protein levels in our pVHL negative RCC lines compared to the pVHL expressing ones, since our results showed decreased protein levels in both, total cell lysates and secreted protein in cell-conditioned media. Therefore, a different pVHL-dependent mechanism might be responsible for the increased levels of TSP-1 in pVHL expressing carcinoma cell lines. Besides the role of pVHL in the proteasome-mediated degradation of HIFα^12^, other pVHL targets such as BIMEL^37^, p53^48^ and Jade-1^38^ have enhanced stabilization and increased half-life. Although this new pVHL function of increasing protein stability could explain the increased TSP-1 levels in pVHL expressing cells, we did not prove pVHL interaction with TSP-1 in pull down assays, this indicating that TSP-1 regulation by pVHL must be indirect. In addition, experiments using different inhibitors of protein degradation did not recover TSP-1 levels in pVHL lacking cells (data not shown). Causes that may account for the loss of TSP-1 might include the diminished positive regulation by tumour suppressor genes like p53, and PTEN among others or an increased negative regulation by oncogenes including myc, Ras, and Id1^49–51^. In this respect, pVHL has been shown as a positive regulator of p53^48^. We found decreased p53 mRNA levels in the 786-O cell line, while no significant differences were observed in the RCC4 cell line (Supplementary Fig. S1a). However, p53 protein levels were significantly decreased in all the analysed pVHL negative and in the pVHL mutants L188V and Y112H (Supplementary Fig. S1b). These results support that VHL-p53 nexus might be involved in the regulation of TSP-1 in these cells. However, Zubac *et al*. have demonstrated no positive correlation between TSP-1 levels and p53 expression in ccRCC^45^. A plausible explanation to this would be that despite p53 levels are higher in pRV-VHL cells, its activity might not be correspondingly increased. Interestingly, the analysis of p21, a p53 target gene, in our ccRCC cells proved a wide variability of results. In the RCC4 cell line, p21 levels were lower compared to RCC4 cells expressing VHL. Accordingly, the levels of p53 were also lower in VHL-deficient cells compared to VHL-expressing RCC4 cells. Conversely, in the 786-O cell line the levels of p21 did not always correlated with those of p53 (Supplementary Fig. S1a,b). This might be related to the phosphorylation state of p53. Interestingly, we observed a higher molecular weight band in p53 blots that is likely to correspond to a phosphorylated form of this protein, especially in 786-O pRV cells and Y112H mutant (Supplementary Fig. S1b). Moreover, precisely in these cells the levels of p21 were higher. These results are difficult to interpret and may explain why other authors found that p53 regulation in ccRCC cells is independent of VHL^52^. Additionally, to test whether TSP-1 regulation in ccRCC cell lines was due to an increase in oncogenes we analysed the levels of myc. However, neither mRNA nor protein myc levels showed a significant increase in pRV or VHL-mutant cells (Supplementary Fig. S1a,b). Other mechanisms such as epigenetic changes, these including hypermethylation of CpG islands around the transcription start site are also involved in the silencing of TSP-1 expression in cancer^31^. On the other hand, DNA methylation on TSP-1 CpG islands was found in approximately a 15% of cases in a cohort of clear cell RCC^53^. However, our results showed no changes at the transcriptional level and therefore this mechanism would not explain the decrease of TSP-1 levels in our RCC cell lines.

Tumour cells may bypass the effects of angiogenic inhibitors either by decreasing the expression of these factors themselves or by increasing the expression of pro-angiogenic factors. TSP-1 supports normal kidney angiostasis, and its loss contributes to the RCC angiogenic phenotype (our current results and results by others)^45–47^. Although the mechanism by which TSP-1 is regulated in ccRCC cells remains elusive, our results clearly contribute to the knowledge of ccRCC, and demonstrate that pVHL loss in these tumours affects their behaviour by modulating TSP-1 expression levels and this may partially explain pVHL anti-tumour activities. Additionally, we demonstrate that pVHL-mediated regulation of TSP-1 is a HIF-independent event. Further studies aiming at elucidating the regulation of angiogenesis inhibitors by pVHL will be beneficial for the development of new anti-angiogenic and anti-tumour therapies for ccRCC.

## Methods

### Cell culture

Renal carcinoma cell line 786-O (CRL-1932) was obtained from the American Type Culture Collection (ATCC: Rockville, MD, USA), and RCC4 and RCC10 cell lines were kindly provided by Dr. W. Kaelin (Dana-Farber Cancer Institute, Boston, MA). 786-O cells were cultured in RPMI 1640 medium with GLUTAMAX-I whereas RCC4 and RCC10 were cultured in DMEM, both supplemented with 10% fetal bovine serum (FBS), 100 units/mL penicillin and 100 μg/mL streptomycin and maintained at 37 °C in a humidified atmosphere of 5% CO_2_. For hypoxia experiments, cells were incubated at 37 °C in an *Invivo*_2_ 400 hypoxia workstation (Ruskinn Technology, West Yorkshire) in the presence of 5% CO_2_ and 1% oxygen for 24 h.

### Retroviral transduction

786-O, RCC4 and RCC10 cells stably expressing pVHL were generated as previously described^54,55^, by retroviral transduction with pRc/CMV or HA-VHL-pRc/CMV vectors, in the text named as pRV or pRV-VHL respectively, provided by Dr. W. Kaelin through Addgene (plasmids #20814 and 19999, respectively). Packaging 293-T cells were grown to 80–90% confluence in 10 cm culture plates. Fresh media without antibiotics was added and mixed with Optimem (1.5 mL/plate), Lipofectamine 2000 (Invitrogen) and the mix of vectors (10 μg of GAG-POL expression vector, 6.5 μg of ENV vector and 8.75 μg of the pRc/CMV or HA-VHL-pRc/CMV vectors). Media was changed after 4–6 h and cells cultured for 48 h. After that time cell media was filtered and mixed with equal volume of fresh medium. Diluted viral supernatants were then added (3 mL/plate) to the cells (786-O, RCC4 and RCC10 pVHL-negative clones) and incubated overnight at 37 °C. The transduction process was repeated two more times and stable clones were maintained in RPMI 1640 medium with GLUTAMAX-I supplemented with 10% FBS, 100 units/mL penicillin, 100 μg/mL streptomycin and 1 mg/ml G418 (Invivogen, San Diego, CA). Additionally, we used 786-O expressing the naturally occurring type 2 C pVHL mutant L188V, or type 2 A pVHL mutant Y112H, that affect its alpha (L188V) or beta domain (Y112H). These mutants were kindly provided by Dr. Michael Ohh (University of Toronto, Canada). Furthermore, to assess the role of HIF in the regulation of TSP-1, retroviral vectors encoding the constitutively active mutated form of HIF-2α [HIF-2α P405A;P531A (P-A)^2^], a mutant lacking transcriptional activity [HIF-2α (P-A)^2^] P405A;P531A;bHLH] or the empty vector pBabe were used^56^.

### Truncated pVHL-mutant generation

Truncated pVHL-forms (1–161, 1–164 and 1–171) were generated by PCR using specific primers for each mutant (Table 1) and human cDNA as template. PCR products were extracted and purified from agarose gels using NucleoSpin gel clean-up kit (Macherey-Nagel) and each fragment was cloned into TOPO Pcr2.1 vector to transform Stbl3 bacteria (Thermo Fisher). Plasmid DNA from ampicillin-resistant clones was extracted with QIAprep Spin Miniprep Kit (QIAGEN) to confirm insert presence by Sanger sequencing (Secugen S.L.). Recombinant DNA was then purified using NucleoBond ® Xtra Maxi Plus (Macherey-Nagel) and amplified fragments were cloned into pLVX lentiviral expression vectors. Lentivirus were produced following a protocol similar to the one used for retroviral transduction. Briefly, packaging 293-T cells were grown to 50–60% confluence in 60 mm culture plates and co-transfected with the following mix of vectors: 12.08 μg of pLVX expression vector, 9.17 μg of psPAX2 vector and 3.67 μg of pVSVg vector. Lentivirus-containing supernatants were collected and added to RCC4 pVHL-negative cells. Stable pVHL-mutants were maintained in RPMI 1640 medium with GLUTAMAX-I supplemented with 10% FBS, penicillin/streptomycin and 1 μg/mL puromycin (Thermo Fisher Scientific).

**Table 1:** List of the primer pairs used for pVHL-mutant generation by PCR.

pVHL mutant	5′-3′ forward primer sequence	5′-3′ reverse primer sequence
**1–161**	ATGCCCCGGAGGGCGGAGAAC	CCGGACAACCTGGAGGCATCACTCTTTCAG
**1–164**	ATGCCCCGGAGGGCGGAGAAC	TAGGCTCCGGACAACCTAGAGGCATCG
**1–171**	ATGCCCCGGAGGGCGGAGAAC	CCTGTAATTCTCAGGCTAGACTAGGCTCCG

### Real Time PCR analysis

Quantitative RNA analysis was performed by real time PCR as previously described^42^. Changes in the *TSP-1*, *VHL*, *VEGF*, *TP53*, *CDKN1A*, *MYC* mRNA levels were analysed, using *HPRT* as housekeeping gene. Cells were grown to 95% confluence in 60-mm culture dishes and total cellular RNA was isolated using the Ultraspec RNA Isolation System (Biotecx Laboratories, Houston, TX). Total RNA (1 μg/sample) was reverse-transcribed to cDNA (Improm II RT, Promega, Madison, WI) in a final volume of 20 μL. For quantitative PCR, 1 μL of cDNA was amplified with the specific primer pairs depicted in Table 2 and the following temperature cycles: 10 min initial denaturation at 95 °C, then 15 s denaturing at 95 °C, 1 min annealing at 60 °C and extended for 40 cycles. Following 40 cycles of DNA amplification, melting curves were performed at 95 °C for 15 s, 60 °C for 1 min and 95 °C for 15 s.

**Table 2:** List of the primer pairs used for gene expression analysis by real-time PCR.

Gene	5′-3′ forward primer sequence	5′-3′ reverse primer sequence
***TSP-1***	ACTGGGTTGTACGCCATCAGG	CTACAGCGAGTCCAGGATCAC
***VHL***	TGACGGACAGCCTATTTTTGC	AACCTGGAGGCATCGCTCTT
***VEGF***	GCTTCCTACAGCACAACAAATGTG	CAGGGATTTTCTTGTCTTGCTCTAT
***TP53***	CGCTTCGAGATGTTCCG	TCAGGCCCTTCTGTCTT
***MYC***	GCTGGATTTTTTTCGGGTAGTG	AGTTCCTGTTGGTGAAGCTAACG
***CDKN1A***	CTGGAGACTCTCAGGGTCGAA	GCGGATTAGGGCTTCCTCTT
***HPRT***	ATTGTAATGACCAGTCAACAGGG	GCATTGTTTTGCCAGTGTCAA

### Conditioned media preparation

For the detection of secreted TSP-1, cells were cultured for 48 hours in DMEM depleted of FBS. Afterwards, conditioned media were collected and complete, EDTA-free, protease inhibitors (Roche, Indianapolis, IN) were added. To discard cell debris, samples were centrifuged (335 g for 5 minutes), 50-fold concentrated and dialyzed to PBS using Amicon Ultra-15, 100 kDa membranes.

### Western-blot analysis

Cells were grown to 95% confluence in 60-mm culture dishes and cell lysates were prepared in reducing 2X Laemmli buffer, boiled at 95 °C for 10 minutes, electrophoretically separated by SDS-PAGE and transferred onto 0.45-µm nitrocellulose membranes (GE Healthcare Life Sciences). Blots were incubated with 5% non-fat dry milk in TBS – 0.1% Tween^TM^ 20 for 30 minutes at room temperature. Afterwards, blots were probed overnight at 4°C with the following primary antibodies: monoclonal anti-TSP-1 antibody Ab-11 (clones D4.6, AG.1, MBC 200.1, Neomarkers Lab Vision), monoclonal anti-pVHL antibody (clone VHL40, Santa Cruz), monoclonal anti-HIF-1α antibody (clone 241809, R&D Systems), polyclonal anti-HIF-2α antibody (Santa Cruz), monoclonal anti p53 antibody (clone DO-1, Santa Cruz), monoclonal anti-myc antibody (clone Y69, abcam) and monoclonal anti-p21 antibody (clone SX118, BD) and monoclonal anti-tubulin antibody (clone DM1A, Sigma). Anti-mouse (Dako) or anti-rabbit (GE Healthcare) HRP-conjugated secondary antibodies were added for 1 hour at room temperature and protein signal was then visualized using Immun-Star WesternC^TM^ kit (BioRad).

### siRNA transfection

Control small interfering RNA (scr) or specific siRNA directed to human TSP-1, HIF-1α, HIF-2α or pVHL (Santa Cruz) were transfected with Lipofectamine 2000 (Invitrogen) according to the manufacturer´s instruction. Three days after transfection, cells were used for the experiments.

### Protein immunoprecipitation

Cells were grown on 150-mm culture plates and lysates were prepared in RIPA buffer (150 mM NaCl, 10 mM Tris HCl, 1% SDS, 1% nonidet P-40, 1% sodium deoxycholate, pH 7.4) with protease and phosphatase inhibitors (Roche). For protein immunoprecipitation, 500 µg of total lysate were pre-cleared with 30 μL of 50% protein G (GE Healthcare, Pittsburgh, PA) in RIPA buffer for 30 minutes. After that, samples were centrifuged (380 g for 3 minutes) and supernatants were incubated at 4 °C for 1 hour with a mixture of protein G and non-specific IgG (Santa Cruz). Samples were again centrifuged (380 g for 3 minutes) and pellets were used as negative control for IP analysis. Conversely, supernatants were incubated with anti-pVHL or TSP-1 antibodies at a final concentration of 1 μL per 100 μg of total protein at 4 °C for 16 hours. Finally, 30 μL of protein G were added and samples were again centrifuged (380 g for 3 minutes), discarding the supernatant. Pellets were rinsed with RIPA buffer and solubilized in reducing 2X Laemmli buffer for immunoblotting.

### Immunofluorescence microscopy

Cells were seeded onto fibronectin coated 13-mm glass coverslips (5 μg/mL fibronectin) and then incubated for 24 h. Afterwards cells were fixed with 4% paraformaldehyde in PBS and permeabilized with 0.5% Triton in PBS with 1% BSA, 100 μg/mL gammaglobulin and 0.05% sodium azide. Cells were blocked for 30 min with 5% BSA in PBS with 100 μg/mL gammaglobulin and 0.05% sodium azide and stained with primary antibodies against β-catenin (BD Transduction Laboratories) or zonulin (ZO)-1 (Thermo Fisher Scientific) followed by Alexa Fluor 488 or 568-labeled secondary antibodies (Life Technologies). Cells were mounted in Prolong Gold (Invitrogen) and imaged with a Leica DMR 020–525.024 fluorescence microscope and Leica immersion objective HCX PL APO 40×/1.25–0.75. Images were collected using Leica TCS software.

### Cell migration and invasion assays

Migration experiments were performed with pVHL positive and negative cells or with cells transfected with a siRNA control or specific siRNA to TSP-1. Cells were plated onto a 24-well tissue culture plate (15 × 10^4^ cells/well) and cultured in DMEM containing 5% (v/v) FBS at 37 °C. Experimental wounds were made with a P200 pipette tip across the central line of the culture plate. Number of migrated cells to the wounded area was determined by phase-contrast microscopy with a Nikon Eclipse TS100 inverted microscope using a Nikon air objective Plan 4×/0.13 and NIS-Elements software, acquiring representative images at representative areas of every experimental condition at time 0 h and after 16 h in culture. Cell migration and invasion were also evaluated in Transwell® filters (6.5-mm diameter, 8 μm pore size, Costar, Corning, NY). RCC cells serum starved for 24 h were seeded on top of the transwells (20 × 10^3^ cells/well in 160 μl RPMI without FBS) and allowed to migrate overnight at 37 °C towards a lower chamber containing RPMI 10% FBS medium or without FBS as a negative control. Non-migrated cells in the upper surface of the membrane were gently removed with Q-tips and cells on the lower surface were fixed and stained with Diff-Quick (International Reagent, Kobe, Japan) and counted under the microscope with a Nikon air objective LWD 20×/0.40. Average of three different random fields per condition was calculated.

### Statistical analysis

All data are presented as mean ± SEM. Two-tailed one-sample t-test was used to compare two groups, and one-way ANOVA test followed by Bonferroni’s or linear trend *post-hoc* tests were used when comparing three or more groups, according with the conditions of normality and homoscedasticity. Shapiro–Wilk and Brown–Forsythe tests were performed to analyse these conditions. *P*-values and *P*-trends < 0.05 were considered significant. All the statistical analyses were performed with GraphPad Prism 7.0a software (San Diego, CA, USA).

## Supplementary information

Supplementary information.

## Data Availability

The datasets generated and/or analysed during the current study are available from the corresponding author on reasonable request.
