# Author Correction: pVHL-mediated regulation of the anti-angiogenic protein thrombospondin-1 decreases migration of Clear Cell Renal Carcinoma Cell Lines

**DOI:** 10.1038/s41598-023-33773-0

**Published:** 2023-05-03

**Authors:** Javier Sevilla-Montero, Raquel Bienes-Martínez, David Labrousse-Arias, Esther Fuertes-Yebra, Ángel Ordóñez, María J. Calzada

**Affiliations:** 1 Biomedical Research Institute La Princesa Hospital (IIS-IP), Department of Medicine, School of Medicine, Autónoma University of Madrid, Madrid, Spain; 2 Research Unit, Hospital Santa Cristina, Biomedical Research Institute Princesa (IIS-IP), Madrid, Spain

Correction to: *Scientific Reports* 10.1038/s41598-020-58137-w, published online 24 January 2020

The Acknowledgements section in the original version of this Article was incomplete. It now reads:

“This work was supported by grants from the Spanish Government (co-funded by European Regional Development Fund, ERDF/FEDER); PI16/02166 from ISCIII, and 2017/EEUU/03 to MJC; Red Temática de Excelencia en Investigación en Hipoxia (SAF 2017-90794-REDT) to MJC. We also thank Dr. Manuel Gómez Gutierrez for critical reading and editing this manuscript.”

The original Article has been corrected.

