# Supplementary material for: pVHL-mediated regulation of the anti-angiogenic protein thrombospondin-1 decreases migration of Clear Cell Renal Carcinoma Cell Lines
Source: Sci Rep. 2020 Jan 24;10:1175. doi: 10.1038/s41598-020-58137-w (PMC6981148; doi:10.1038/s41598-020-58137-w)

Supplementary Information

pVHL-mediated regulation of the anti-angiogenic protein thrombospondin-1 decreases migration of Clear Cell Renal Carcinoma Cell Lines.

Javier Sevilla-Montero[1*], Raquel Bienes-Martínez[1*], David Labrousse-Arias[1], Esther Fuertes-Yebra[2], Ángel Ordóñez[2], and María J. Calzada[1].

[1]*Biomedical Research Institute La Princesa Hospital (IIS-IP), Department of Medicine, School of Medicine, Autónoma University of Madrid, Madrid, Spain,* [2]*Research Unit, Hospital of Santa Cristina, Biomedical Research Institute Princesa (IIS-IP) Madrid , Spain.*

***Contributed equally to this work**

Address correspondence to: Maria J. Calzada, PhD. Biomedical Research Institute La Princesa Hospital (IIS-IP). Autónoma University of Madrid. Diego de Leon, 62. 28006 Madrid, Spain

Email: mariajose.calzada@uam.es

Phone: (+34-915202371)

Supplementary Figure S1: Determination of p53, myc and p21 levels in 786-O and RCC4 ccRCC cells. (a) Quantitative RT–PCR analysis was performed to determine *TP53*, *MYC* and *CDKN1A* mRNA expression levels from pVHL-positive (pRV-VHL), pVHL-negative (pRV), L188V and Y112H pVHL-mutant-expressing 786-O cells, and pVHL-positive and negative RCC4 cells. mRNA levels from at least three independent experiments are expressed as fold change over pVHL-positive cells, normalized with *HPRT* as housekeeping gene and presented as mean ± SEM. Statistical comparisons between different conditions were made using either one-way ANOVA test followed by Bonferroni's *post-hoc* test or one-sample t-test (n.s. = non-significant, *$P < 0.05$, **$P < 0.01$, ***$P < 0.005$). **(b)** Protein levels from control pVHL-positive, pVHL-negative, L188V and Y112H pVHL-mutant-expressing 786-O cells, and pVHL-positive and negative RCC4 were determined by western blot probed against p53, myc, pVHL, p21 and α-tubulin as loading control. Representative images and band quantifications by densitometry of p53, myc and p21 from at least three independent experiments are shown and presented as mean ± SEM. Statistical comparisons between different conditions were made using either one-way ANOVA test followed by Bonferroni's *post-hoc* test or one-sample t-test (n.s. = non-significant, *$P < 0.05$, **$P < 0.01$, ***$P < 0.005$). Full-length blots are presented in Supplementary Figure S2.

a

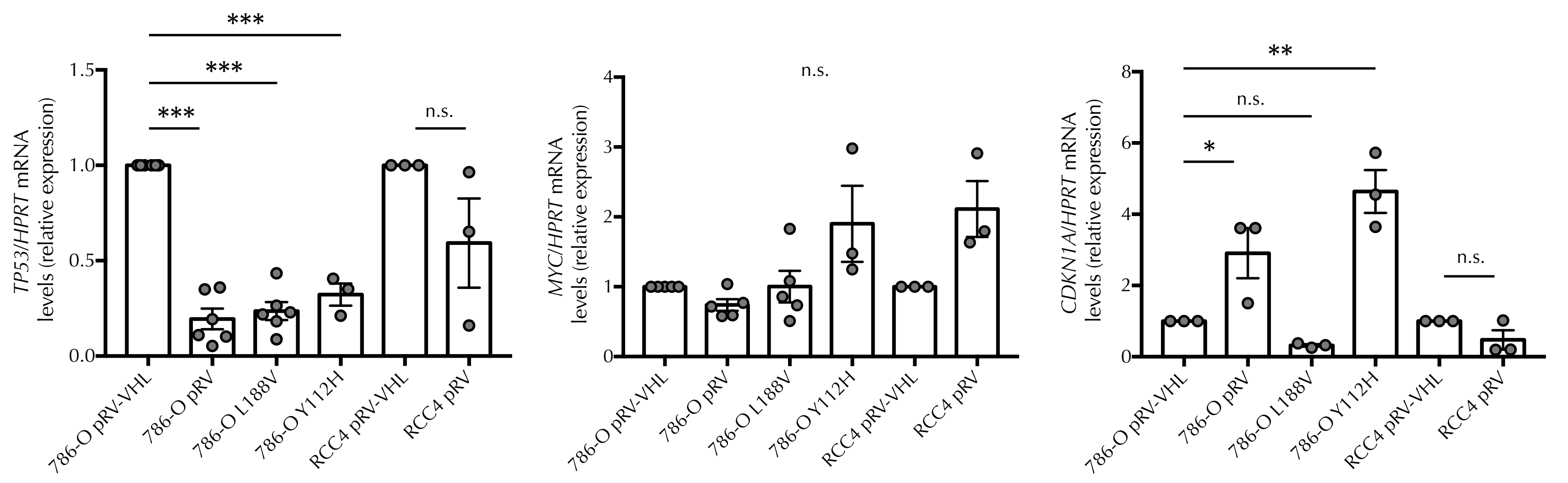

b

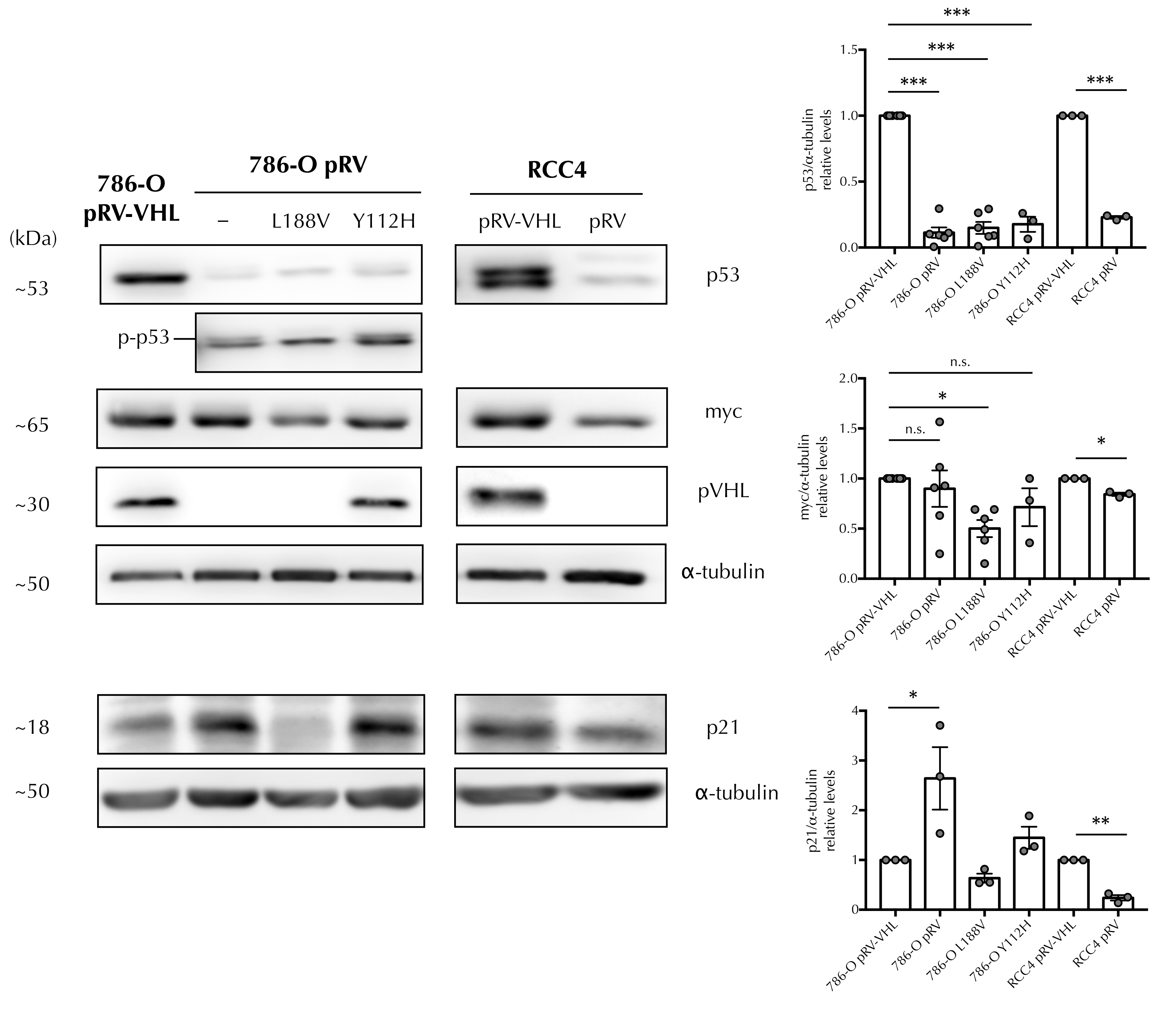

Supplementary Figure S1

Supplementary Figure S1: Determination of p53, myc and p21 levels in 786-O and RCC4 ccRCC cells. (a) Quantitative RT–PCR analysis was performed to determine *TP53, MYC* and *CDKN1A* mRNA expression levels from pVHL-positive (pRV-VHL), pVHL-negative (pRV), L188V and Y112H pVHL-mutant-expressing 786-O cells, and pVHL-positive and negative RCC4 cells. mRNA levels from at least three independent experiments are expressed as fold change over pVHL-positive cells, normalized with *HPRT* as housekeeping gene and presented as mean ± SEM. Statistical comparisons between different conditions were made using either one-way ANOVA test followed by Bonferroni's *post-hoc* test or one-sample t-test (n.s. = non-significant, *$P < 0.05$, **$P < 0.01$, ***$P < 0.005$). **(b)** Protein levels from control pVHL-positive, pVHL-negative, L188V and Y112H pVHL-mutant-expressing 786-O cells, and pVHL-positive and negative RCC4 were determined by western blot probed against p53, myc, pVHL, p21 and α-tubulin as loading control. Representative images and band quantifications by densitometry of p53, myc and p21 from at least three independent experiments are shown and presented as mean ± SEM. Statistical comparisons between different conditions were made using either one-way ANOVA test followed by Bonferroni's *post-hoc* test or one-sample t-test (n.s. = non-significant, *$P < 0.05$, **$P < 0.01$, ***$P < 0.005$). Full-length blots are presented in Supplementary Figure S2.

Supplementary Figure S2: Full-length gels

Full-length gels for Figure 1

b

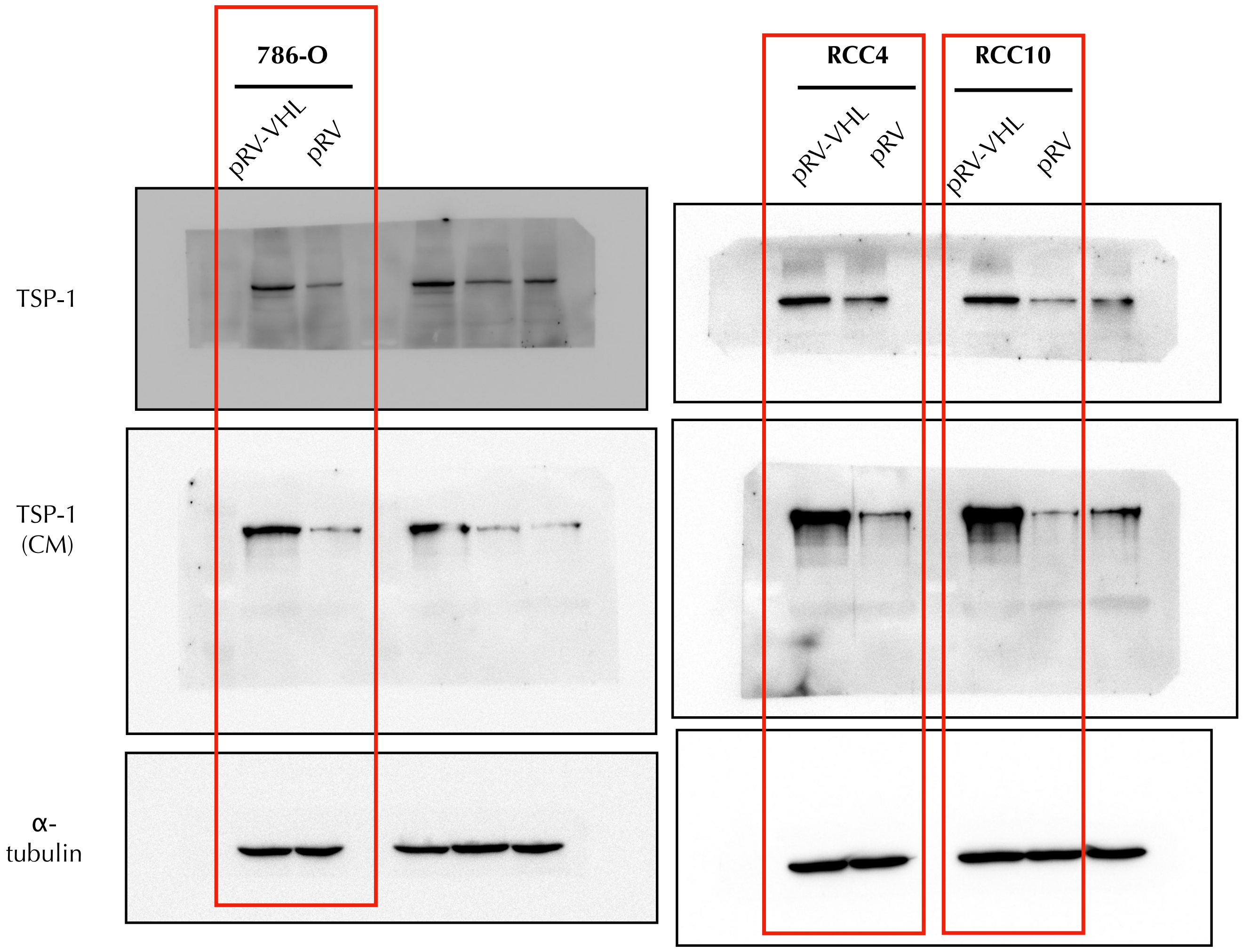

c

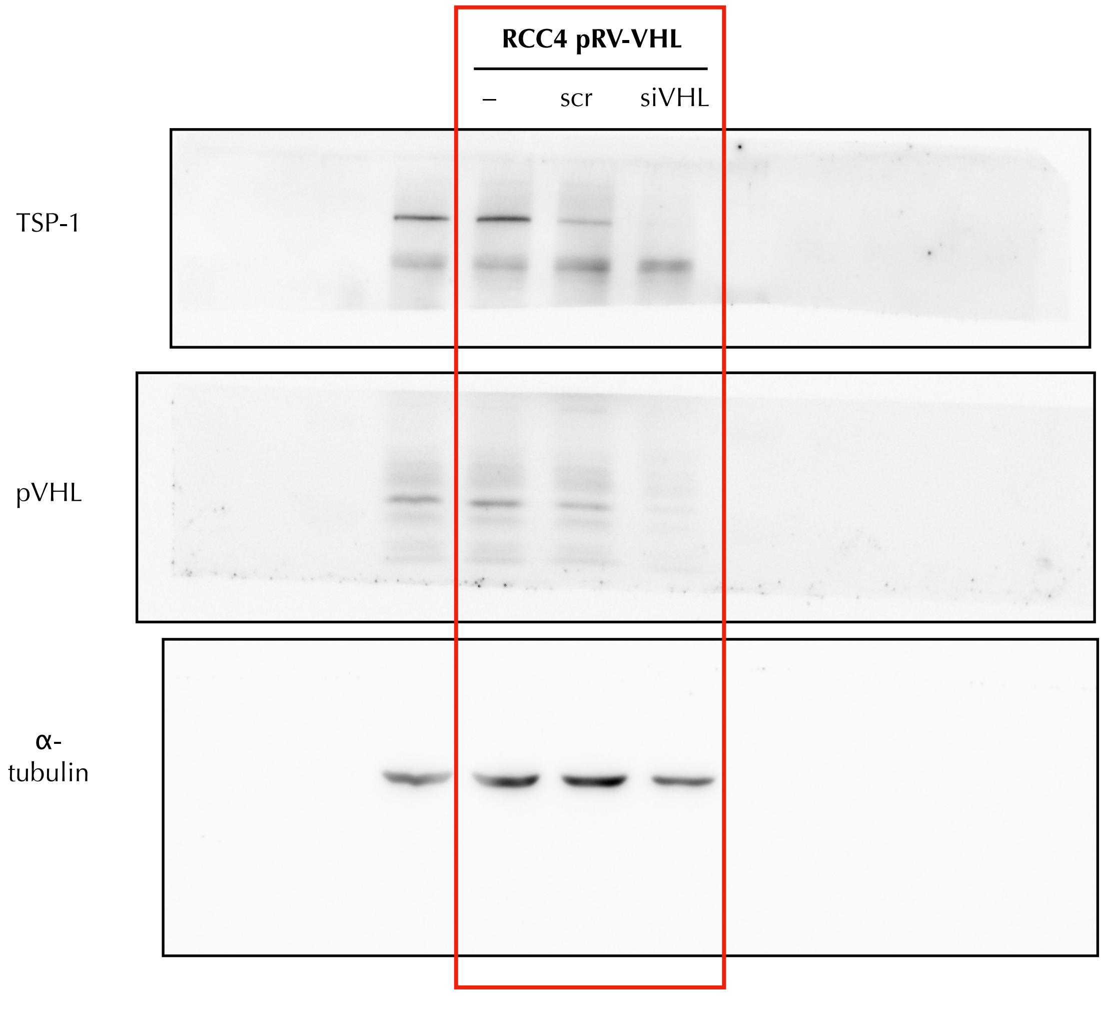

Full-length gels for Figure 2

a

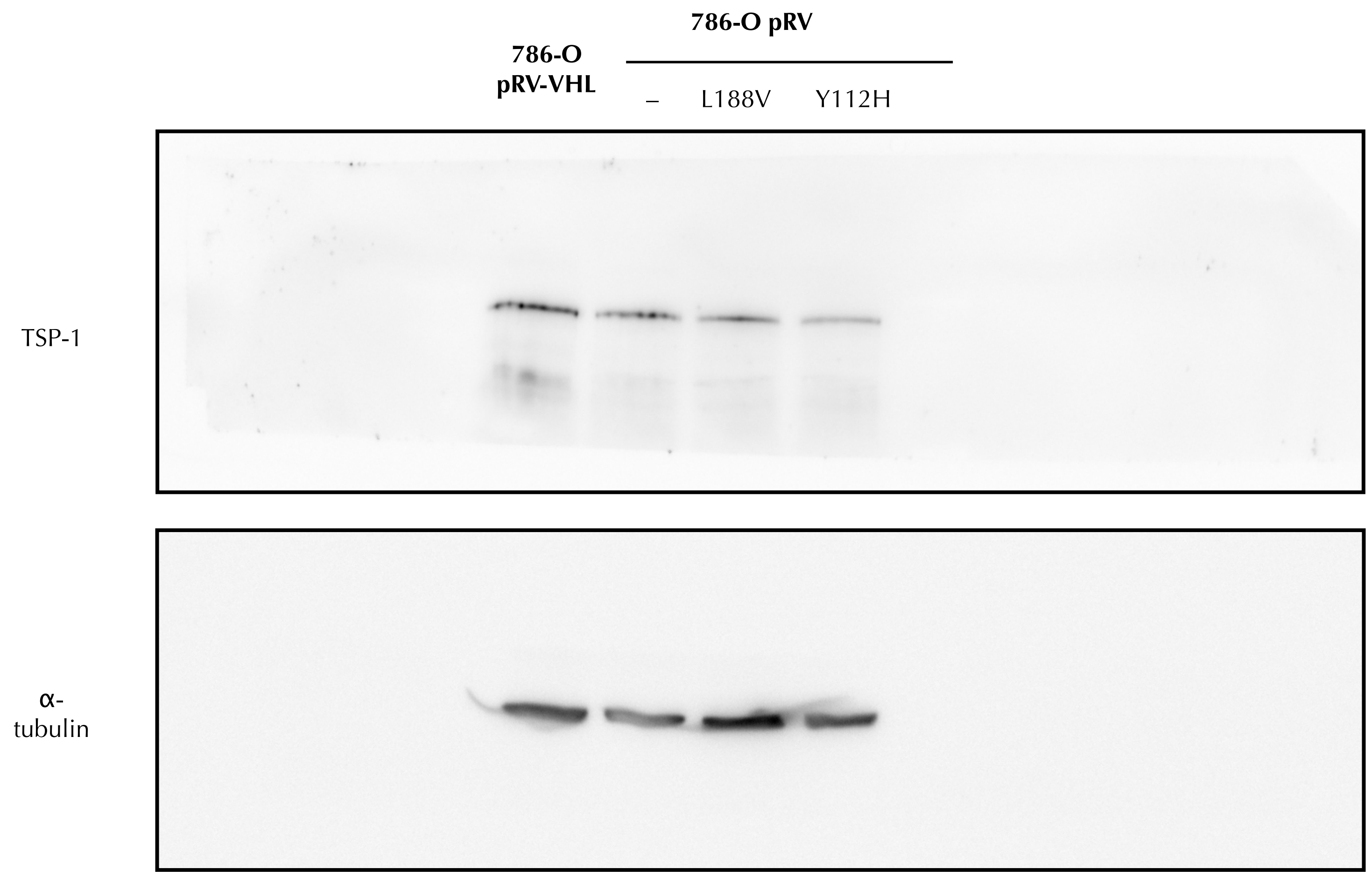

b

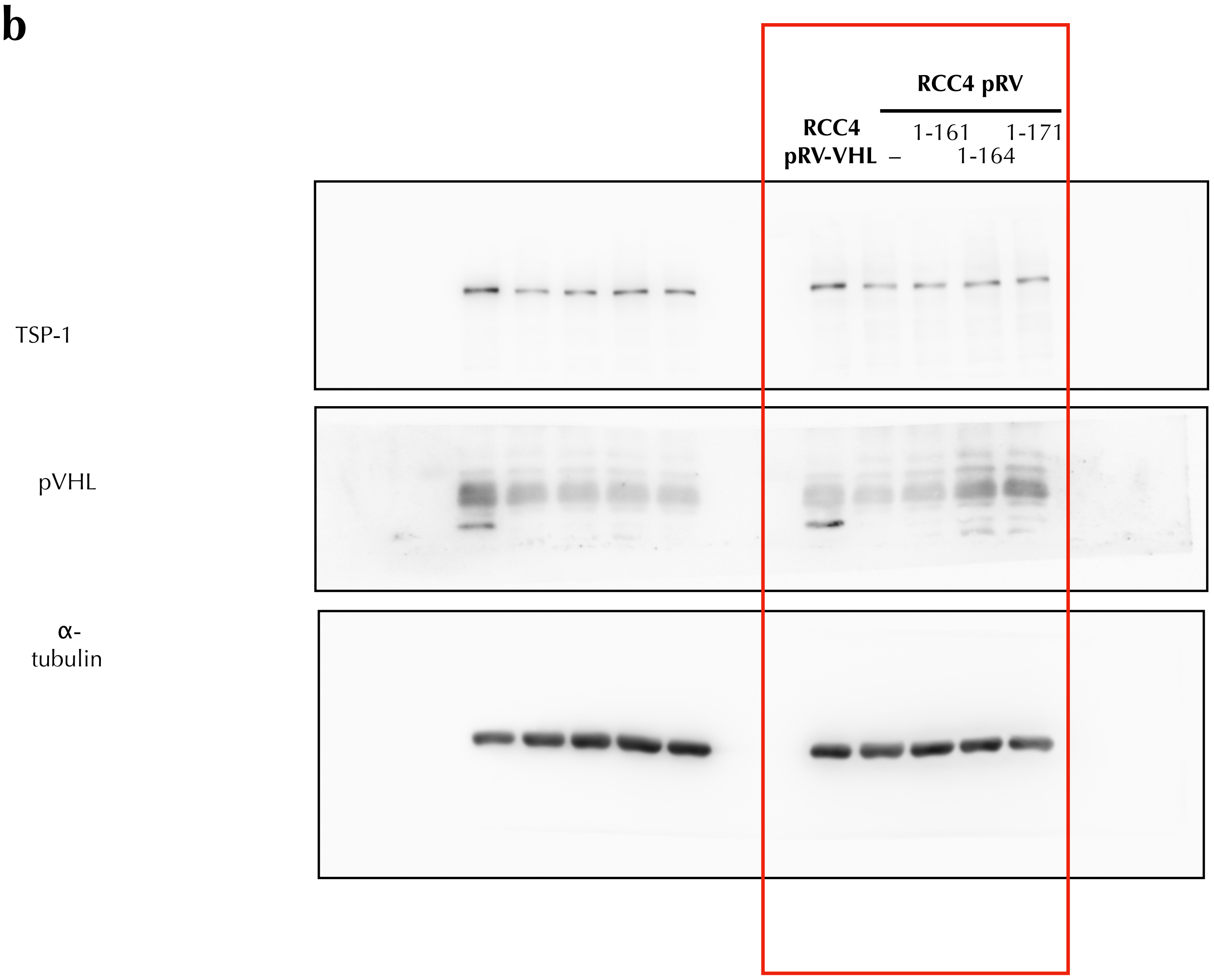

Full-length gels for Figure 3

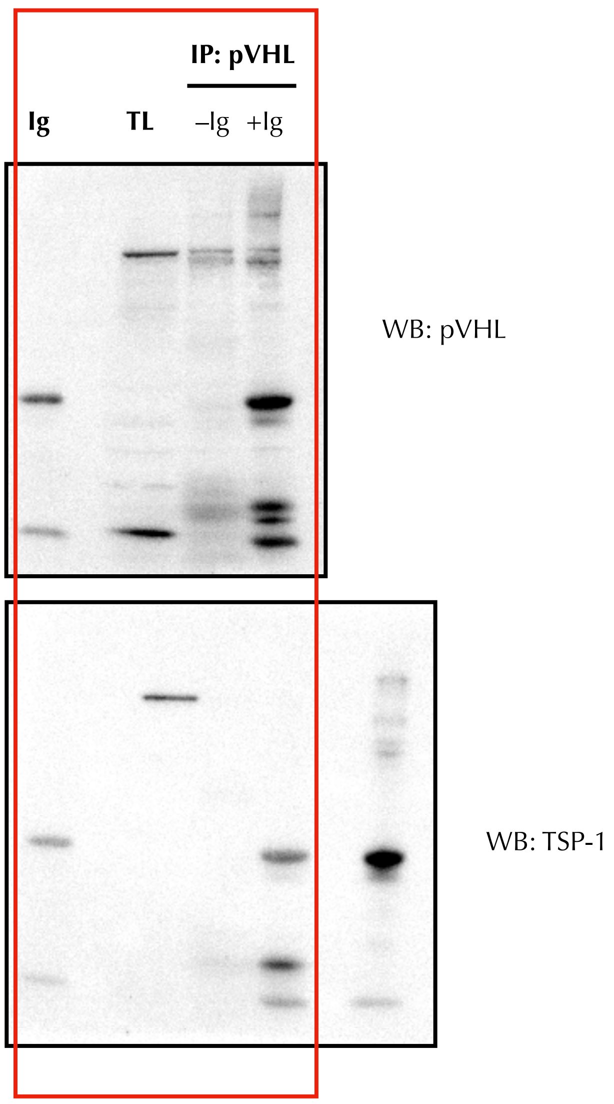

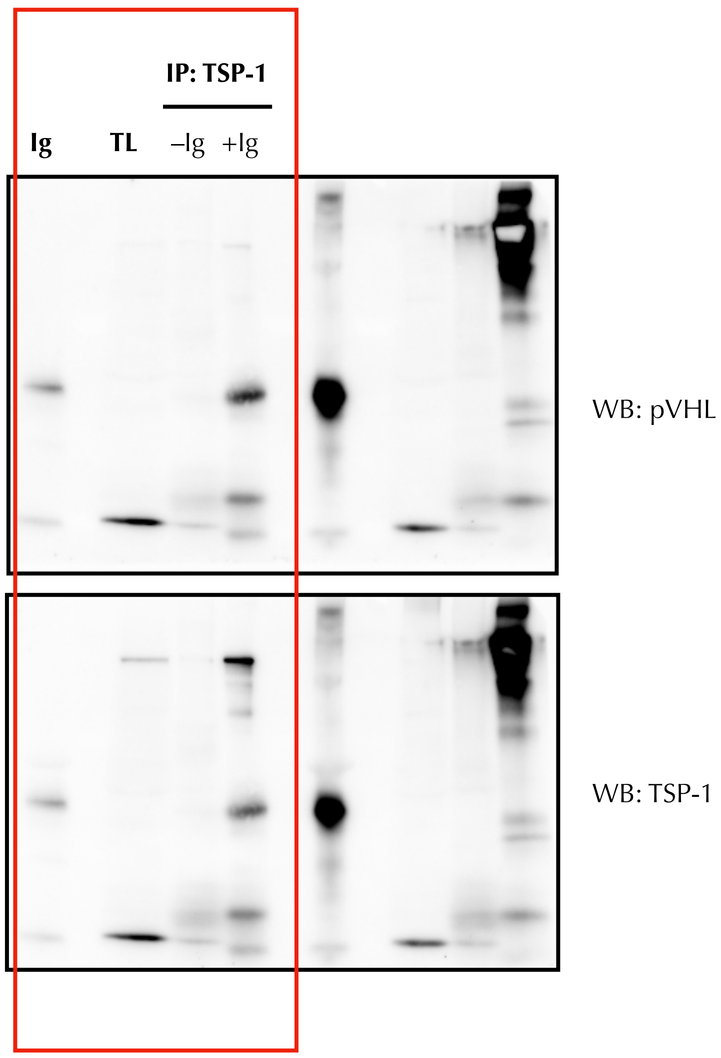

Full-length gels for Figure 4

b

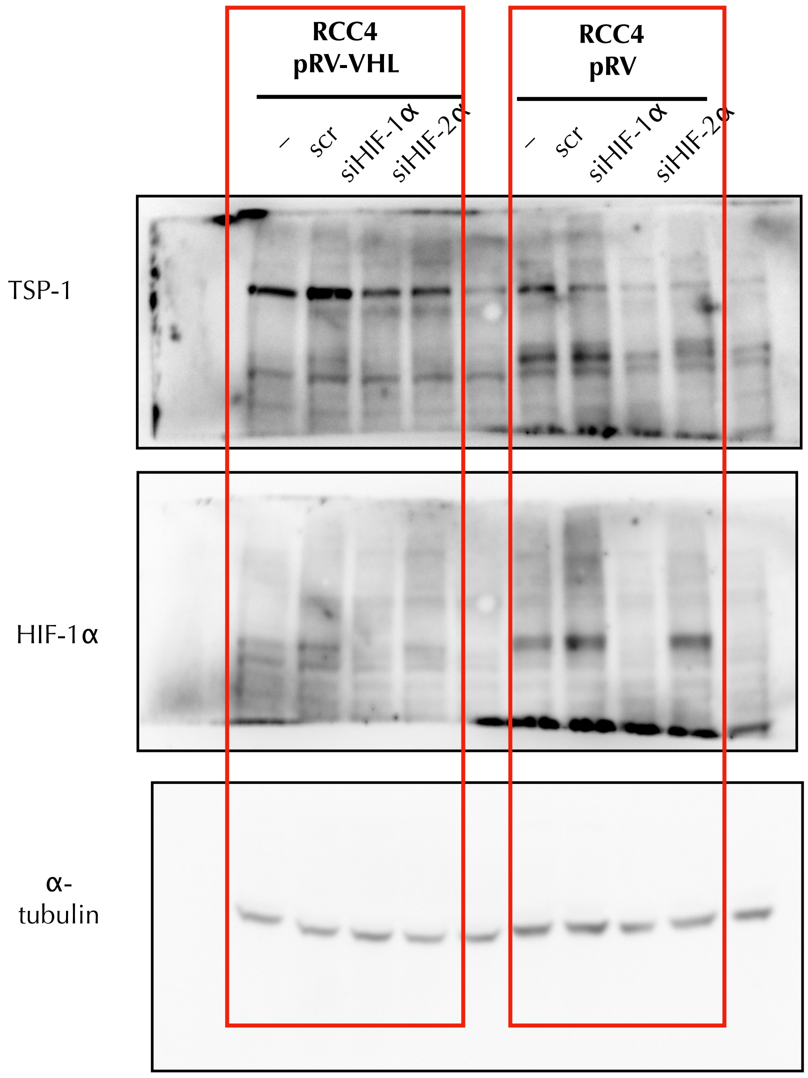

c

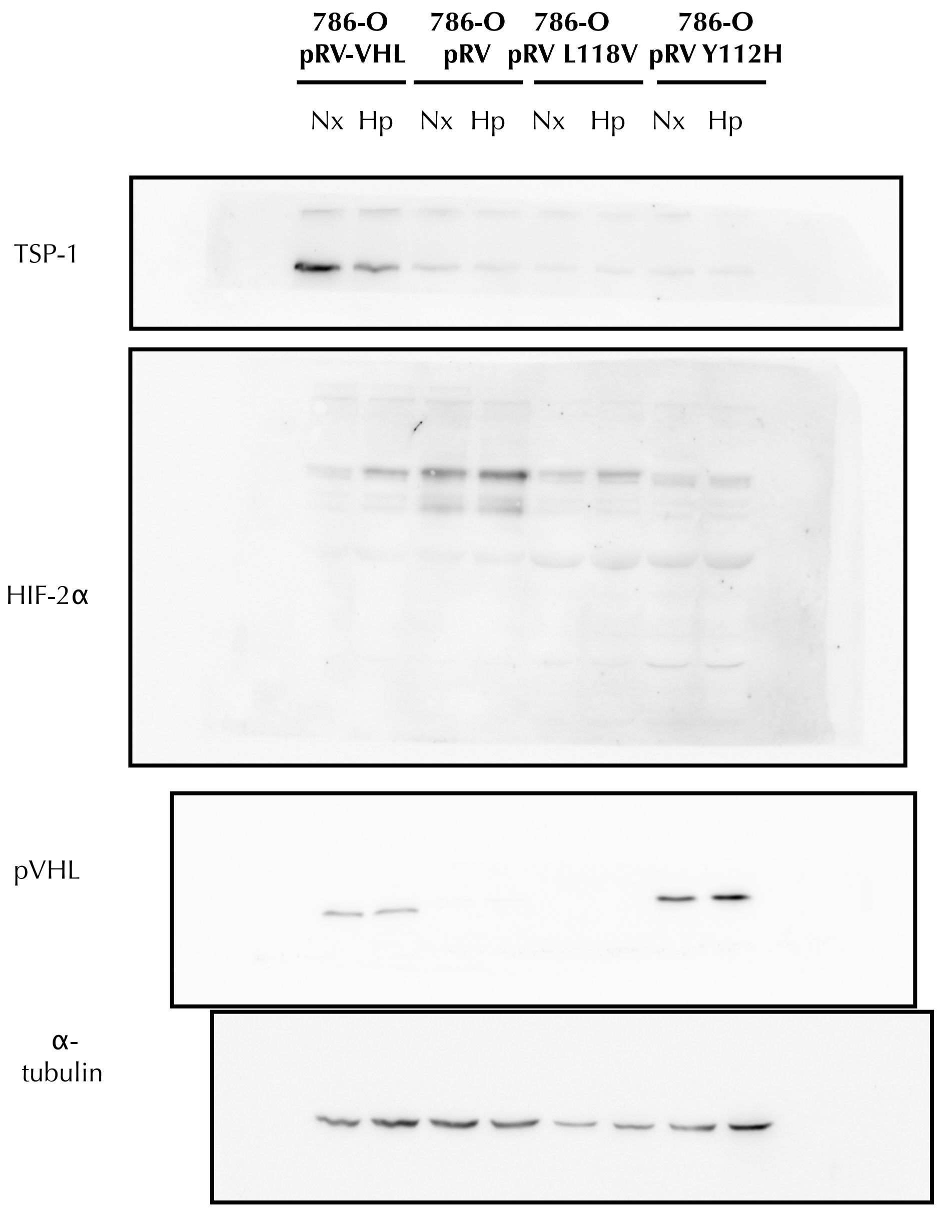

Full-length gels for Figure 5

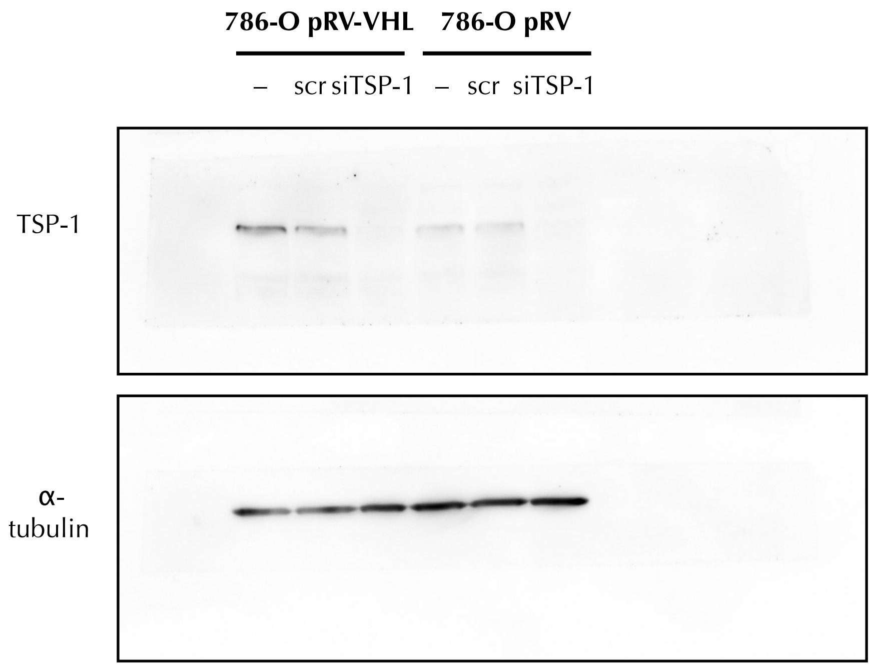

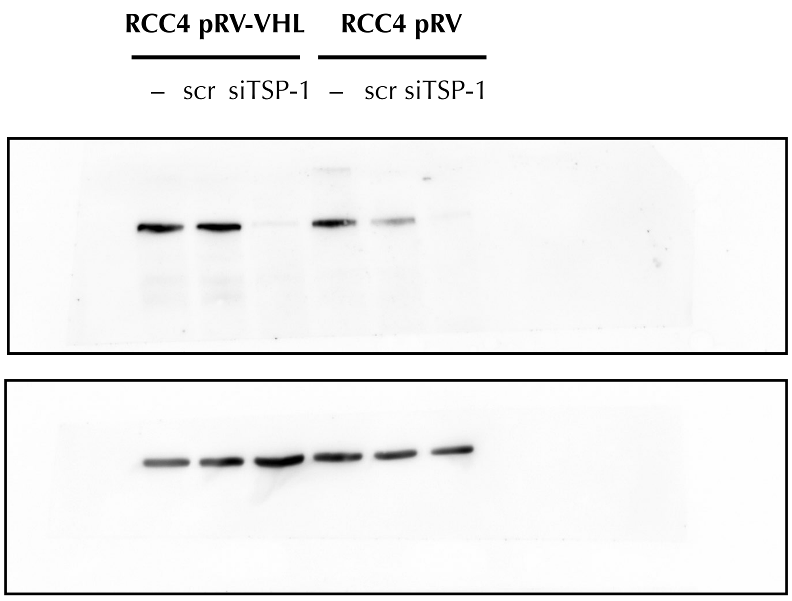

Full-length gels for Supplementary Figure S1

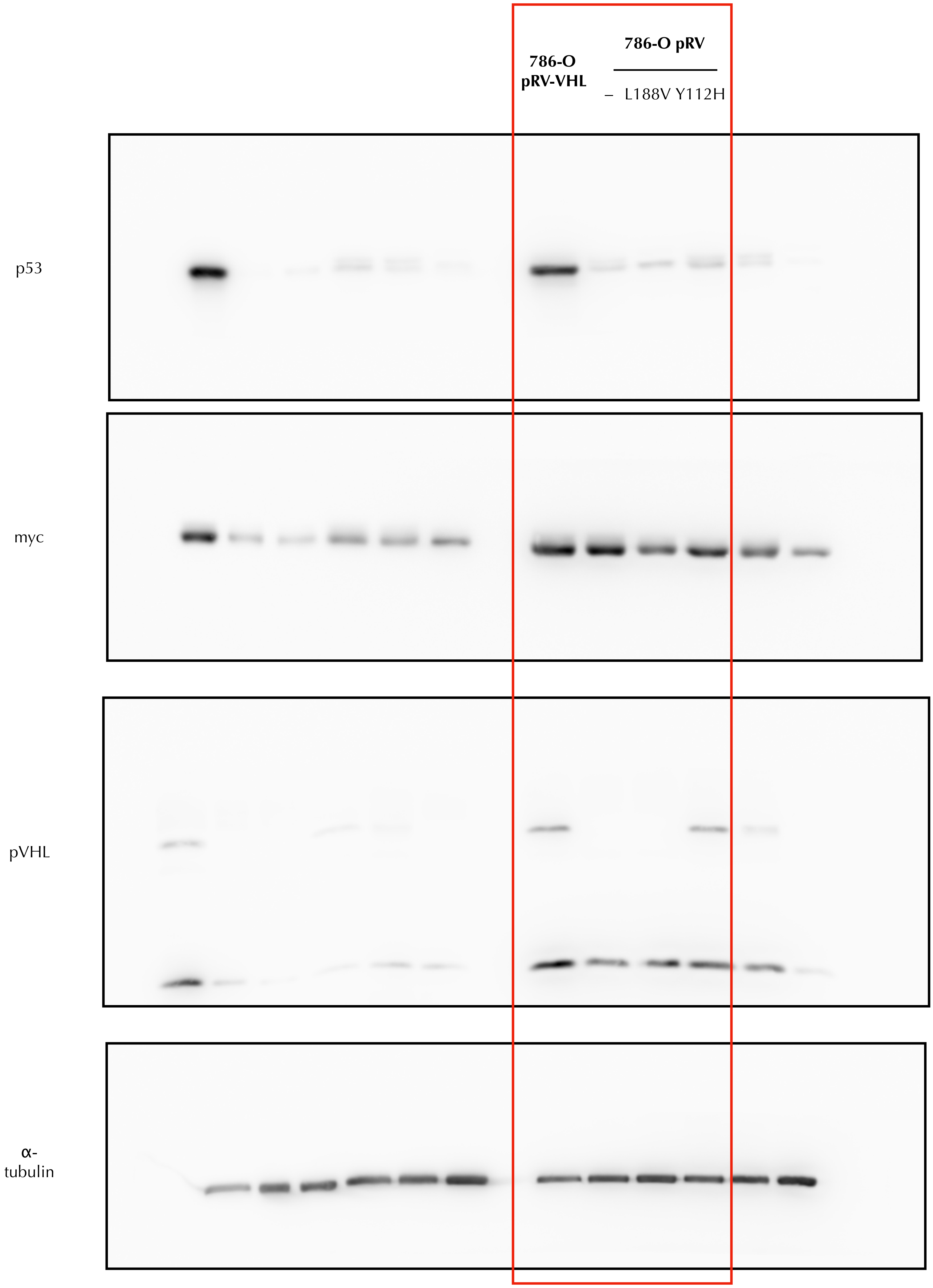

Full-length gels for Supplementary Figure S1

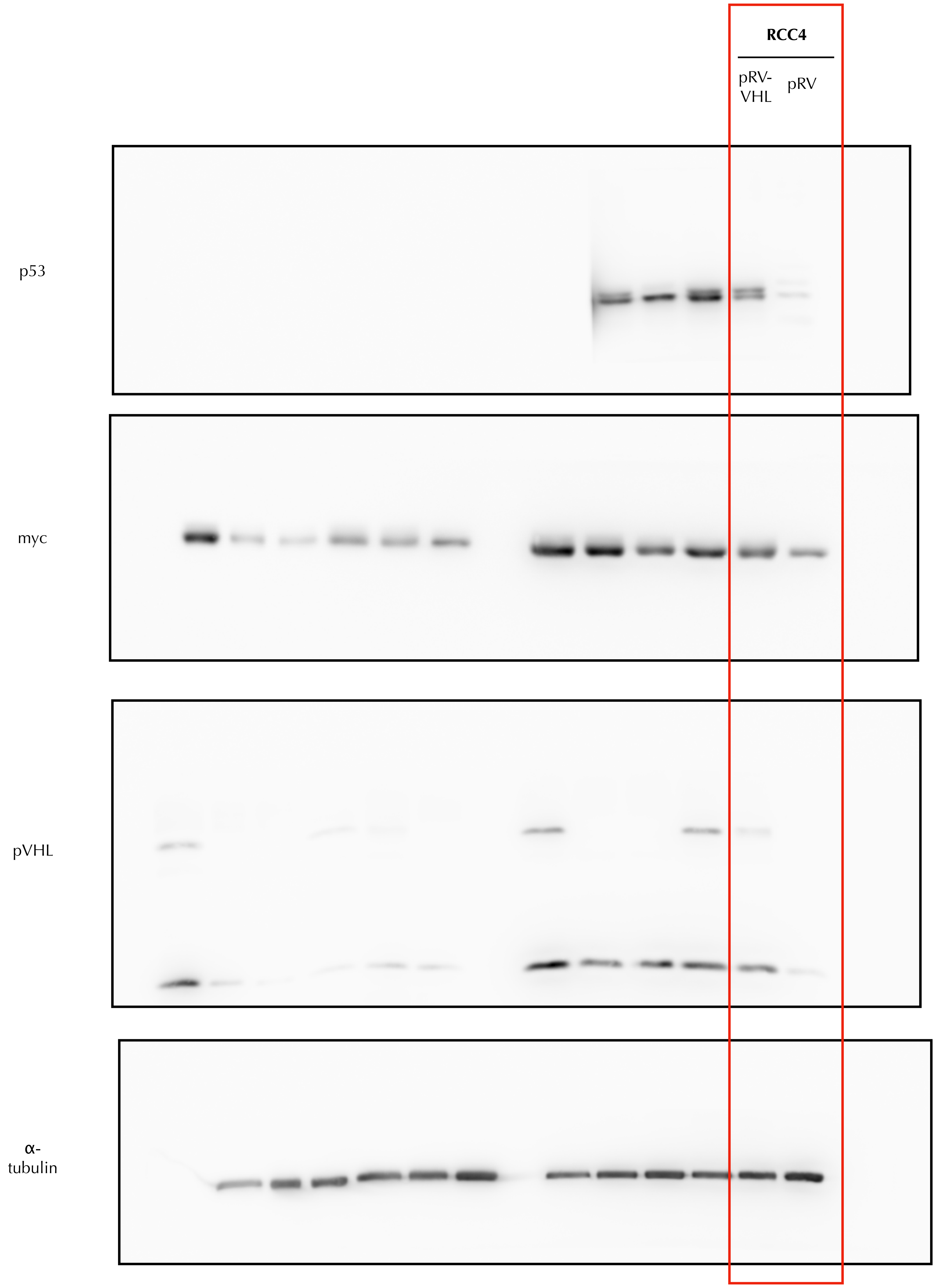

Full-length gels for Supplementary Figure S1

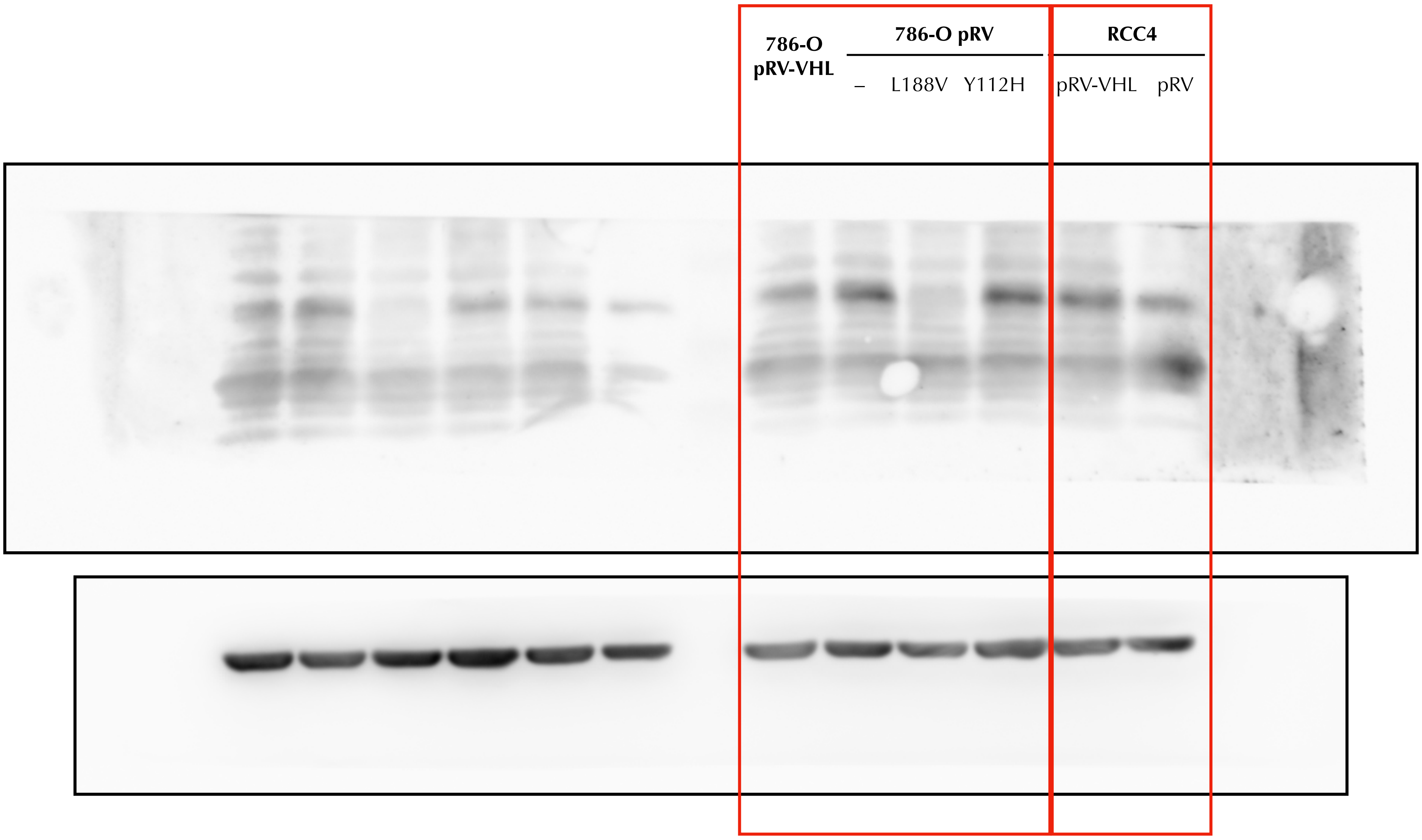

Supplement: Supplementary file 1 — Supplementary information. [file 41598_2020_58137_MOESM1_ESM.pdf]
